# Morphology and Molecular Composition of Purified Bovine Viral Diarrhea Virus Envelope

**DOI:** 10.1371/journal.ppat.1005476

**Published:** 2016-03-03

**Authors:** Nathalie Callens, Britta Brügger, Pierre Bonnafous, Hervé Drobecq, Mathias J. Gerl, Thomas Krey, Gleyder Roman-Sosa, Till Rümenapf, Olivier Lambert, Jean Dubuisson, Yves Rouillé

**Affiliations:** 1 Univ. Lille, CNRS, Inserm, CHU Lille, Institut Pasteur de Lille, U1019—UMR 8204—CIIL—Center for Infection and Immunity of Lille, Lille, France; 2 Heidelberg University Biochemistry Center, INF 328, Heidelberg, Germany; 3 Institut de Chimie et Biologie des Membranes et des Nano-objets, CNRS UMR-5248, Université de Bordeaux, Pessac, France; 4 Univ. Lille, CNRS, Institut Pasteur de Lille, UMR 8161—M3T—Mechanisms of Tumorigenesis and Target Therapies, Lille, France; 5 Institut Pasteur, Unité de Virologie Structurale, Département de Virologie, Paris, France; 6 CNRS UMR 3569, 25–28 Rue du Docteur Roux, Paris Cedex 15, France; 7 Institute of Diagnostic Virology, Friedrich-Loeffler-Institut (FLI), 17493 Greifswald–Insel Riems, Germany; 8 Institute of Virology, University of Veterinary Medicine, Vienna, Austria; Purdue University, UNITED STATES

## Abstract

The family *Flaviviridae* includes viruses that have different virion structures and morphogenesis mechanisms. Most cellular and molecular studies have been so far performed with viruses of the Hepacivirus and Flavivirus genera. Here, we studied bovine viral diarrhea virus (BVDV), a member of the Pestivirus genus. We set up a method to purify BVDV virions and analyzed their morphology by electron microscopy and their protein and lipid composition by mass spectrometry. Cryo-electron microscopy showed near spherical viral particles displaying an electron-dense capsid surrounded by a phospholipid bilayer with no visible spikes. Most particles had a diameter of 50 nm and about 2% were larger with a diameter of up to 65 nm, suggesting some size flexibility during BVDV morphogenesis. Morphological and biochemical data suggested a low envelope glycoprotein content of BVDV particles, E1 and E2 being apparently less abundant than E^rns^. Lipid content of BVDV particles displayed a ~2.3 to 3.5-fold enrichment in cholesterol, sphingomyelin and hexosyl-ceramide, concomitant with a 1.5 to 5-fold reduction of all glycerophospholipid classes, as compared to lipid content of MDBK cells. Although BVDV buds in the endoplasmic reticulum, its lipid content differs from a typical endoplasmic reticulum membrane composition. This suggests that BVDV morphogenesis includes a mechanism of lipid sorting. Functional analyses confirmed the importance of cholesterol and sphingomyelin for BVDV entry. Surprisingly, despite a high cholesterol and sphingolipid content of BVDV envelope, E2 was not found in detergent-resistant membranes. Our results indicate that there are differences between the structure and molecular composition of viral particles of Flaviviruses, Pestiviruses and Hepaciviruses within the *Flaviviridae* family.

## Author Summary

Bovine viral diarrhea virus (BVDV) is the etiologic agent of mucosal disease and bovine viral diarrhea, two economically important diseases of the livestock. BVDV is a member of the Pestivirus genus in the *Flaviviridae* family, which also includes Hepacivirus and Flavivirus genera. Members of this family share similar genome organization and replication strategies, but differ about their mode of transmission and particle structure. Whereas most studies have been so far performed on viruses of the Hepacivirus and Flavivirus genera, little is known about infectious particles of pestiviruses. In this study, we set up a novel purification method of BVDV infectious particles and analyzed their morphology by cryo-electron microscopy and their molecular composition by mass spectrometry. Our results provide new insights into the structure and biochemical composition of a pestivirus infectious particle, and have implications for research on molecular mechanisms of their morphogenesis and entry.

## Introduction

The *Flaviviridae* family includes important human and animal pathogens. Members of this family are enveloped, positive-stranded RNA viruses that share similarities in replication and genome organization. They have been classified into 4 genera, namely Flavivirus, Hepacivirus, Pestivirus and Pegivirus. The Flavivirus genus consists of a large number of arthropod-borne viruses. The Hepacivirus genus includes hepatitis C virus (HCV) and recently identified closely related viruses. Members of the Pestivirus genus are animal pathogens including bovine viral diarrhea virus (BVDV), classical swine fever virus (CSFV) and border disease virus (BDV) of sheep [1]. The Pegivirus genus contains a few HCV-related viruses, formerly known as GB-viruses.

Despite similarities in genome organization and replication mechanisms, members of this family have very different modes of transmission: most flaviviruses are transmitted by mosquitoes or ticks, while the mode of transmission of pestiviruses can be oro-nasal and diaplacental and that of HCV is parenteral. These differences in transmission mode are mirrored by differences in the structure of infectious particles and envelope proteins of flaviviruses and hepaciviruses. Flavivirus infectious virions are ~50 nm particles fully coated with 90 dimers of class II envelope protein E [2,3]. In contrast, HCV particles have been proposed to display a lipoprotein-like structure [4,5] containing high amounts of apolipoproteins, especially apoE [6,7].

These different structural organizations denote differences in morphogenesis mechanisms. Envelope proteins appear to be a major driving force of flaviviruses morphogenesis, yielding both infectious particles and non-infectious, capsid-less, sub-viral particles. In contrast, the release of sub-viral particles from pestivirus-infected cells has not been reported. Capsid-less particle release by HCV-infected hepatocytes has been reported [8]. However this most probably reflects the incorporation of E1E2 envelope glycoproteins in apoB-containing lipoproteins [9], rather than the production of flavivirus-like sub-viral particles. Indeed, HCV appears to contain a limited number of envelope glycoproteins per virion [6,7], precluding any role in driving the budding process. HCV morphogenesis is proposed to be aided by the machinery of VLDL formation of hepatocytes [4]. This results in the production of infectious particles of low buoyant density reflecting a high content in neutral lipids cholesteryl ester [6] and probably also triacylglycerol. Remarkably, recent reports also indicate that the structures of envelope proteins E2 of BVDV and HCV are radically different from each other and from class II proteins of flaviviruses [10–13].

For pestiviruses, much less is known concerning the structure of the virions and the mechanisms of morphogenesis [14]. Electron microscopy images of BVDV-infected cells have revealed the presence of small enveloped ~50 nm viral particles of irregular form [15] budding in the ER membrane [16]. Pestivirus virions produced in cell culture have buoyant densities of 1.10–1.13 g/cm^3^ in sucrose gradients [17–19], intermediate between those of flaviviruses (1.19–1.23 g/cm^3^) and HCV (1.05–1.10 g/cm^3^) [1]. Moreover, it has recently been demonstrated that core, the capsid protein, is not essential for the release of infectious CSFV particles [17]. All these observations suggest that pestiviruses have a virion structure and morphogenesis mechanisms that differ from those of HCV and flaviviruses. To address this question, we set up a method of purification of BVDV virions and analyzed their morphology by cryo-electron microscopy and their protein and lipid content by mass spectrometry.

## Results

### Purification of BVDV particles

**Fig 1:**
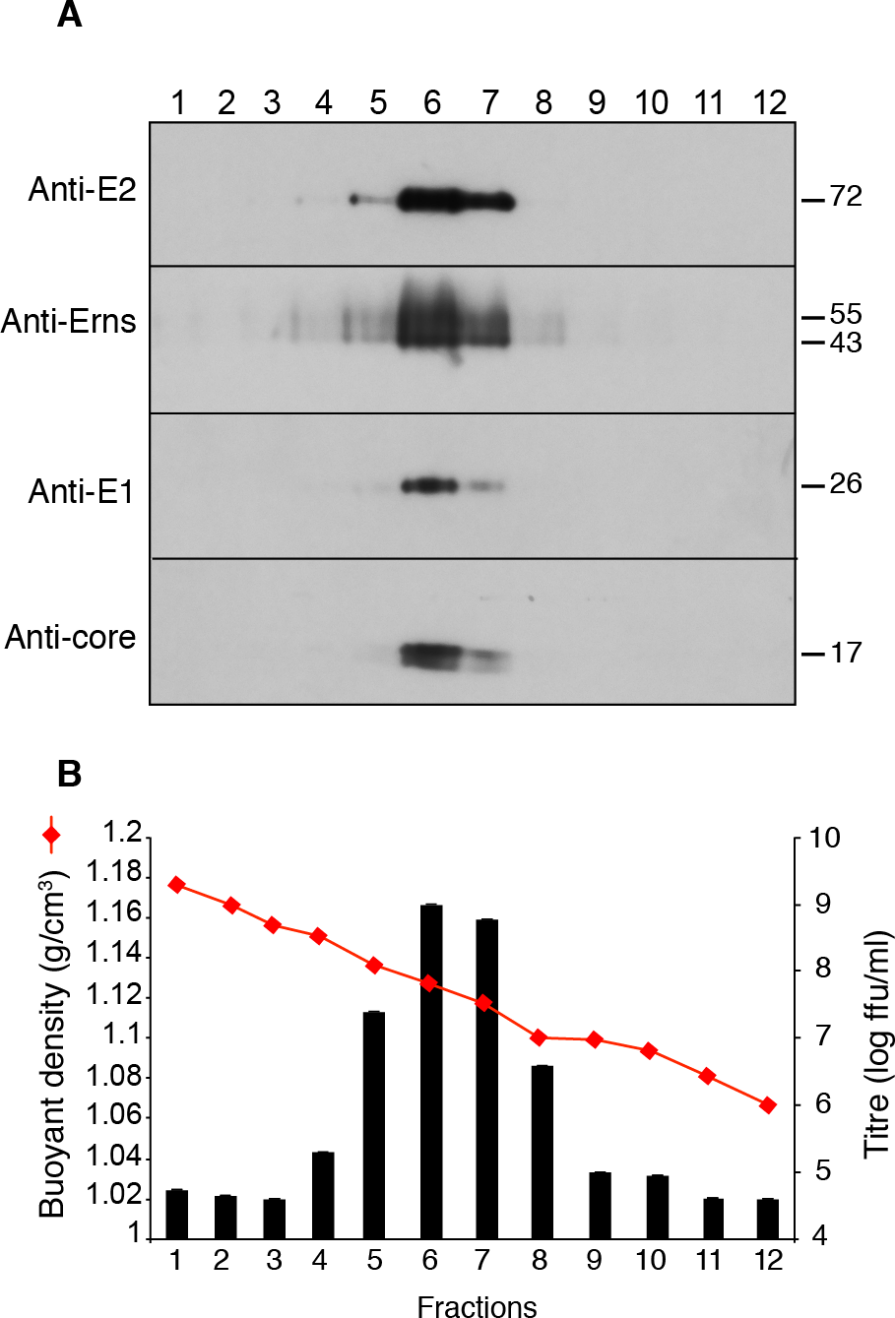
Flotation of BVDV particles in a sucrose density gradient. BVDV was collected from culture medium of infected MDBK cells, concentrated by PEG precipitation, isolated in a 15–30% sucrose interface, adjusted to 40% sucrose, overlaid with a 5–35% sucrose density gradient and centrifuged. Fractions were collected from the bottom of the tube. (A) Immunoblot analysis of gradient fractions with indicated antibodies under reducing conditions for C, E1 and E^rns^, and non-reducing conditions for E2. (B) Infectious titers and buoyant density of gradient fractions.

Viral particles were purified from culture medium of MDBK cells infected with a non-cytopathic strain of BVDV in order to reduce the presence of apoptotic bodies and other cell-derived debris, which are released in high amounts during an infection with a cytopathic strain. Four steps were required in order to obtain a purified virus. Viral particles were precipitated with PEG and separated from heavy membranes and from soluble material by centrifugation through two cushions of 15 and 30% sucrose. The infectious material was found in a fraction located just below the 15–30% interface. The virus was further purified by flotation in a sucrose density gradient. Viral proteins were detected by immunoblotting and the infectivity of each fraction of the gradient was measured. A single peak of C, E^rns^, E1 and E2 proteins was detected in the gradient (Fig 1A), which matched the distribution of infectious particles (Fig 1B). E1 and E2 were detected as single bands, whereas E^rns^ appeared as a smear or multiple bands, likely representing heterogeneity of E^rns^-associated glycan structure. C was detected as two bands. However, it is not clear if this actually represents post-translational modification or limited degradation of the protein, because the doublet was not consistently observed. The buoyant density of this peak was measured as 1.125 ± 0.006 g/cm^3^ (n = 5, mean ± SD). This value is consistent with previously reported buoyant density values of infectious BVDV particles from partially purified or unpurified sources [18,19]. Finally, most of the remaining membranous contaminants were removed with a chromatography step on cellulose-sulfate beads. At each purification step, viral particles were quantified both by infectious titer and core immunoblotting. Typically, starting from about 900 ml of culture medium titrating approximately 10^6^−10^7^ ffu/ml, this method yielded 50 μl of purified virus with a titer in the range of 10^10^−10^11^ ffu/ml.

### Analysis of purified BVDV particles by cryo-electron microscopy

**Fig 2:**
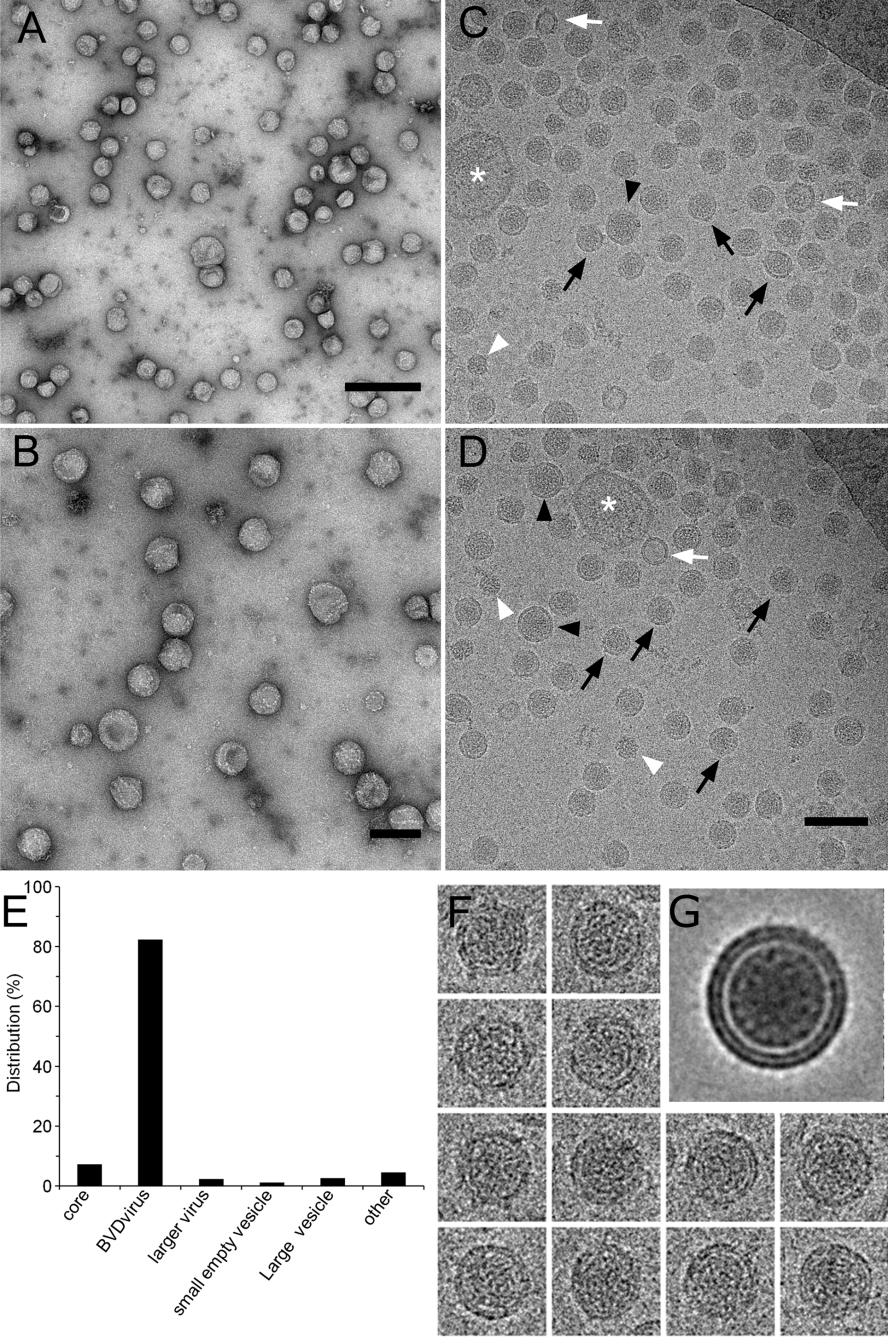
Analysis of purified BVDV particles by electron microscopy. Visualization of purified BVDV particles after negative staining (A, B), or by cryo-electron microscopy (C, D). Black arrows: enveloped, capsid-containing particles of ~50 nm, black arrowheads: larger enveloped, capsid-containing particles, white arrowheads: non-enveloped, capsid-like particles, white arrows: small empty vesicles, white asterisks: large vesicles. (E) Quantification of different objects indicated in C and D. (F) examples of images used for virion reconstruction. (G) Virion reconstructed by projecting 160 images of 50-nm particles. Bars, 200 nm (A) or 100 nm (B,D).

Examination of purified fractions by electron microscopy after negative staining revealed the presence of numerous particles of about 50 nm and a few contaminating objects (Fig 2A). At higher magnification, 50-nm particles appeared more or less spherical (Fig 2B). For a better preservation of BVDV morphology, purified particles were observed by cryo-electron microscopy, a technique that preserves the hydration state of the sample and avoids dehydration artifact induced by negative staining. Again, the more abundant objects were spherical particles with a diameter of about 50 nm (Fig 2C and 2D and S1 Fig). These objects were absent from samples purified from uninfected cells (S2 Fig). They were bounded by two electron-dense thin layers with a clear layer in between, which likely represent the lipid bilayer of the viral envelope. The inner part of the viral particles was made of electron-dense material, likely corresponding to the viral capsid. The preparation also contained some smaller (30–35 nm) non-enveloped objects of the same size and morphology as viral capsids, a small number of larger (55–65 nm) virus-like particles and other unrelated objects (Fig 2C and 2D).

We could not find any clear morphological evidence for the presence of envelope glycoproteins at the surface of viral particles using cryo-electron microscopy. We performed immunogold labeling of purified BVDV under negative staining condition, but surprisingly very few viral particles were labeled (S3 Fig). To verify that anti-E2 antibodies actually recognize E2 glycoproteins present on purified viral particles, we analyzed their neutralization potency. No significant difference of neutralization curves (IC50 = 3.44 ng/ml and 3.53 ng/ml for unpurified and purified BVDV, respectively) was observed between unpurified and purified BVDV (S4 Fig), suggesting that E2 glycoproteins were not altered during purification.

**Fig 3:**
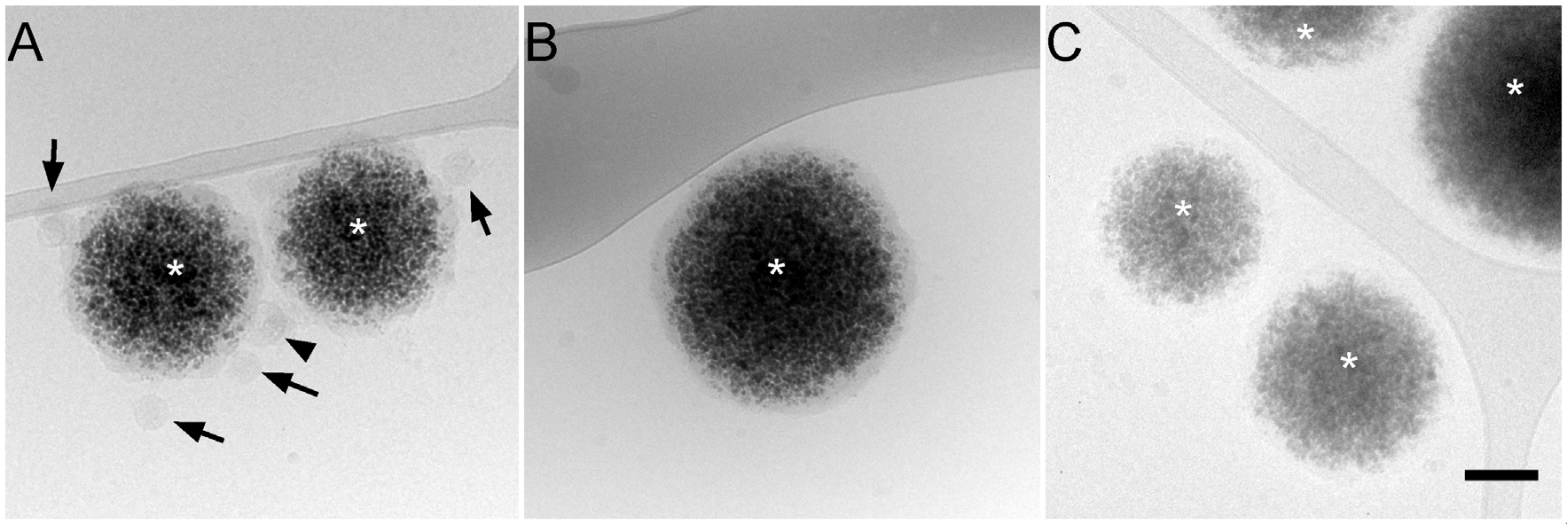
Immunocapture of BVDV particles. Protein G-coated magnetic beads (asterisks) were incubated with anti-BVDV E2 mAbs (A), no antibody (B), or an irrelevant mAb (C) and purified BVDV fraction and observed by cryo-electron microscopy. Black arrows: enveloped, capsid-containing particles of ~50 nm, black arrowheads: larger enveloped, capsid-containing particles. Bar, 100 nm.

To confirm that the 50-nm particles actually are BVDV virions, purified material was incubated with an antibody to BVDV glycoprotein E2 coupled to magnetic beads and the immunocaptured material was visualized by cryo-electron microscopy. Magnetic beads covered with anti-E2 antibodies captured 50-nm virus-like particles, thus confirming that these objects are BVDV virions exposing E2 glycoprotein at their surface (Fig 3A). Occasionally, larger virus-like particles could also be observed (Fig 3A), suggesting that they are viral particles of larger size. Control beads with no antibody (Fig 3B) or with an irrelevant antibody (Fig 3C) did not bind any particles. This result indicates that the 50-nm particles, as well as larger particles of the same morphology expose BVDV E2 glycoprotein at their surface and thus confirms that these particles actually are BVDV virions. Accordingly, these objects were not observed in control fractions purified from non-infected cells (S2 Fig), and very few of them were found in the flow-through fraction of the chromatography step, which is enriched in contaminating material (S5 Fig). Importantly, the contaminating material principally consisted of vesicles that are structurally different from core-containing 50-nm particles of the purified fraction.

The purity of a virus preparation was evaluated by counting objects visible in cryo-electron microscopy according to their morphology. Examples of objects that were counted are indicated in Fig 2C and 2D. Among 1871 objects counted, 82% were 50-nm enveloped virions, 7% non-enveloped core-like particles and 2% larger virions; 1% of the material was made of small vesicles about the same size as a virion with no internal core and 2% were larger vesicles. Finally 6% were unrelated objects (Fig 2E). This suggests that this purified fraction contained at least 82% of viral particles, if one considers only the 50-nm enveloped particles, and up to 91% of viral particle-related objects, if one considers small non-enveloped objects as virions that have lost their envelope during the final purification step, and larger virus-like particles as viruses containing a larger capsid. This indicates a residual contamination of about 9%.

In order to have a more defined view of BVDV particles, 160 images of viral particles with a diameter of 50 nm were aligned. A few of them are shown in Fig 2F. The projection resulting from this alignment is shown in Fig 2G. The average image clearly showed the lipid bilayer and the internal capsid structure. As observed on individual viral particles, there was no evidence of spikes or of any other proteins at the membrane surface. Moreover, the lipid bilayer of the envelope was clearly visible as two distinct electron-dense layers on most particles (Fig 2C, 2D and 2F and S1 Fig) and on the average image (Fig 2G), suggesting that this membrane is poor in trans-membrane proteins, as observed on HIV virions [20] or HCV pseudoparticles [21].

We also tried to purify infectious particles of cytopathic strain NADL using the same protocol. However, the final pellet mainly contained contaminating vesicles of different sizes and shapes and very few 50-nm capsid-containing virus-like BVDV particles (S6 Fig). Therefore this purification protocol is probably not suited for purifying BVDV particles from cytopathic strains, which generate a large number of membranous contaminants during infection.

To try to better define NADL particles from contaminating vesicles, we treated purified NADL particles at pH 5.1 in the presence of a reducing agent. Such a treatment has been shown to induce fusion of BVDV bound to the surface of cells [22]. However, no change of morphology was observed (S7 Fig), suggesting that the structure of envelope proteins were not modified, or that they were present at low density on the virions and therefore difficult to observe.

### Dimerization of envelope proteins

**Fig 4:**
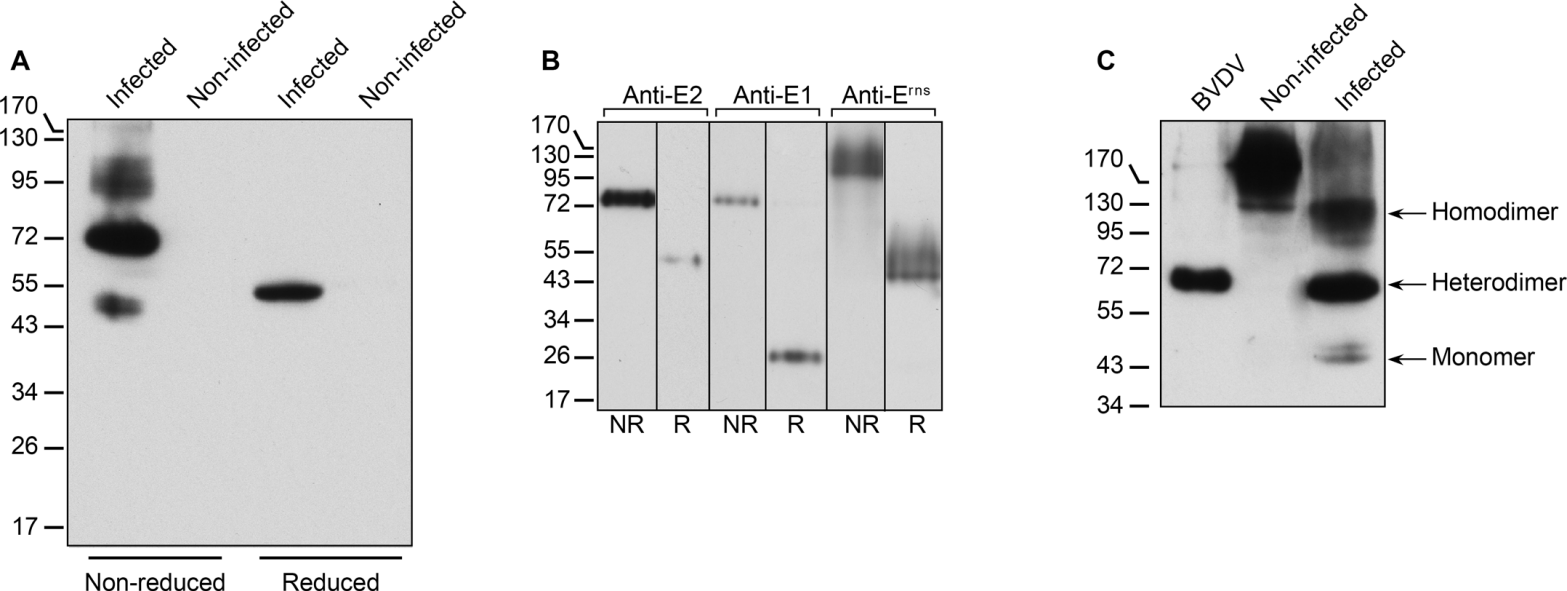
Envelope glycoprotein dimerization. (A) Triton-X100 lysates of infected and non-infected MDBK cells analyzed by immunoblot with a mix of anti-E2 mAbs WB214 and WB166 under reduced or non-reduced conditions. (B) Purified BVDV analyzed by immunoblot with anti-E2 mAbs WB214 and WB166, anti-E1 mAb 8F2 or anti-E^rns^ pAb under reduced (R) or non-reduced (NR) conditions. (C) Lysates of purified BVDV and infected MDBK cells containing similar amounts of E1E2 heterodimer, and a lysate of non-infected MDBK cells were analyzed by immunoblot with anti-E2 mAbs under non-reduced condition. The bands of E2 monomer, E2 homodimer and E1E2 heterodimer are indicated.

Pestiviruses are endowed with envelope glycoproteins that are engaged in covalent homo- and heterodimers [23,24]. During the purification, the detection of a single E2-immunoreactive band of about 75 kDa in non-reduced virus samples (Fig 1A) suggested the presence of virion-associated covalent E1E2 heterodimers, but no band corresponding to E2 monomer (~55 kDa) or covalent homodimer (~110 kDa) could be observed. To assess whether our anti-E2 antibody could detect E2 monomers and homodimers, we probed a lysate of infected MDBK cells under reducing and non-reducing conditions. Three bands were detected with sizes of about 55, 75 and 110 kDa, consistent with the presence of E2 monomer, covalent E1E2 heterodimers and covalent E2 homodimers respectively in infected MDBK cells (Fig 4A). None of these bands was detected in non-infected cells lysate. Only one band was detected in reduced sample (Fig 4A). This band had an apparent size close to that of the 55-kDa band detected under non-reducing condition, confirming its being E2 monomer. A slight difference of migration of E2 monomer under reducing and non-reducing conditions was observed, as previously reported [23]. This likely reflects changes in the conformation of the glycoproteins induced by the reducing agent. A more compact conformation of the native protein, resulting from the presence of intramolecular disulfide bridges, would explain its faster migration. These data confirmed that the anti-E2 antibodies we used are able to detect E2 monomer, homodimers and heterodimers.

Purified virus samples were probed under reducing and non-reducing conditions. Anti-E1 and anti-E2 antibodies detected a band of the same size (~75 kDa) under non-reducing condition (Fig 4B). Under reducing conditions, an anti-E1 antibody revealed a band of 25 kDa, the expected size of E1 monomer, and an anti-E2 antibody revealed a band of 55 kDa, which is the size of E2 monomer observed in cell lysates. This confirmed that the 75-kDa band is a covalent E1E2 heterodimer. On the other hand, we detected bands of different size and aspect with anti-E^rns^ antibody than with E1 and E2 antibodies in non-reduced samples, indicating the absence of covalent E1E^rns^ or E2E^rns^ heterodimers. Single bands of ~90–100 kDa and ~45–50 kDa were detected under non-reducing and reducing conditions, respectively, consistent with the presence of covalent E^rns^ homodimer in pestivirus virions, as reported previously [25,26].

To further assess the potential presence of E2 homodimer and E2 monomer on BVDV particles, lysates of partially purified BVDV (flotation gradient fraction) and of infected MDBK cells containing similar amounts of E1E2 heterodimer were analyzed by immunoblot under non-reducing condition. No band corresponding to E2 homodimer or E2 monomer could be observed even after prolonged exposure of the blot (Fig 4C). Scanning and quantification of the bands indicated that the amounts of E2 homodimer and monomer on BVDV particles are less than 0.3% of E1E2 heterodimer.

In contrast, E2 homodimer was detected in all steps during the purification of NADL particles (S8 Fig). It is not clear if this difference resulted from the difference of viral strain or if E2 homodimer was contributed by the large amounts of cell-derived vesicles generated by this cytopathic strain of BVDV. Nevertheless, the variation of homodimer:heterodimer ratio during the purification rather suggests that homodimer and heterodimer were probably present on different populations of objects, probably including both virions and contaminating vesicles.

### Protein content of purified BVDV particles

**Fig 5:**
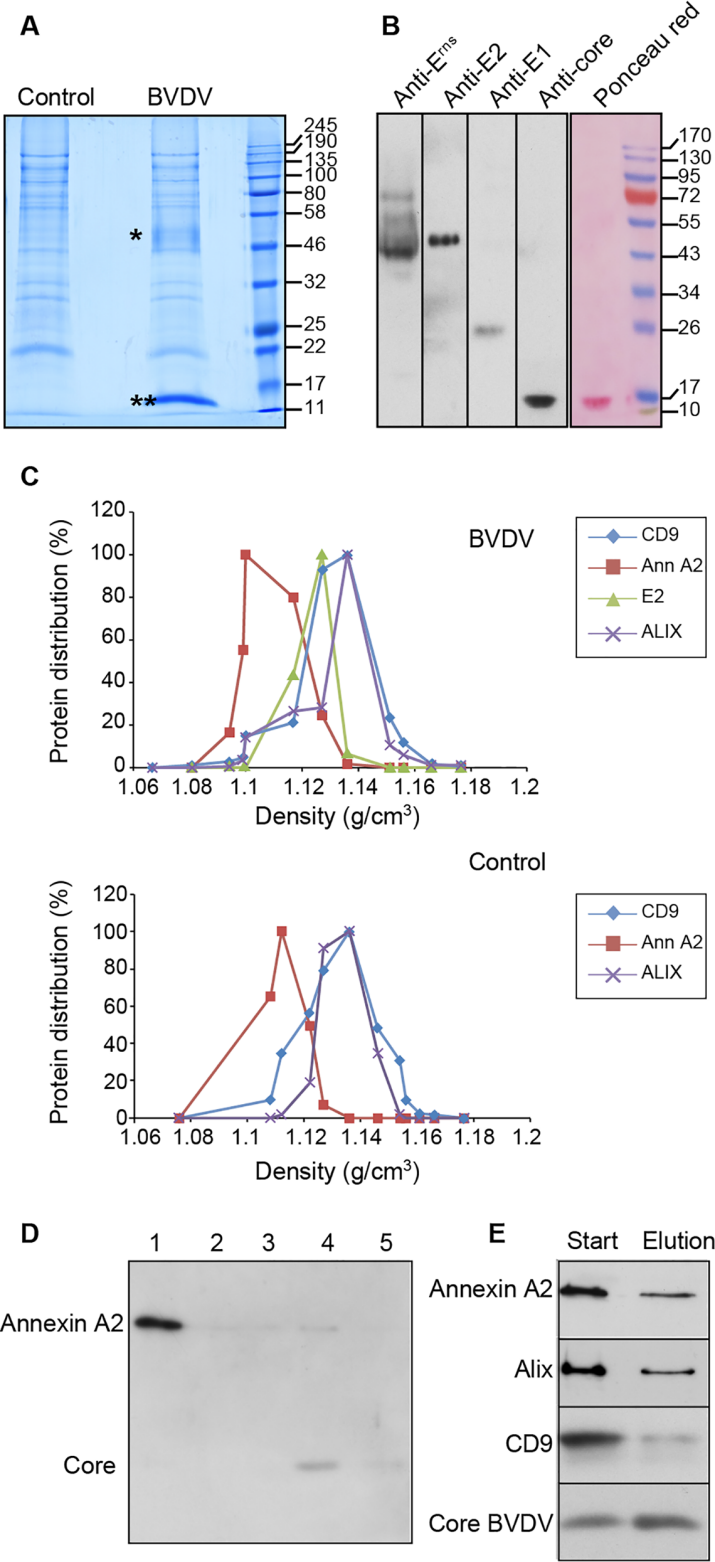
Protein content of purified BVDV fraction. (A) A purified BVDV fraction and an equivalent fraction obtained from non-infected cells (control) were analyzed by SDS-PAGE under reducing conditions and colloidal Coomassie blue staining. Asterisk, E^rns^, double asterisk, core. (B) Immunoblot analysis under reducing conditions and Ponceau red staining of purified BVDV fraction. (C) Distribution of CD9, annexin A2, ALIX and BVDV E2 in sucrose density gradient of BVDV (top) and control (bottom) fractions. Bands were quantified by densitometry and plotted over fraction density. (D) Immunoblot analysis of annexin A2 and BVDV core in flow through (1), PBS washes (2 and 3) and 0.4M NaCl elution (4 and 5) fractions of cellulose-sulfate column. 1:50 of each fraction was loaded on the gel. (E) Immunoblot analysis of Annexin A2, ALIX, CD9 and core in BVDV fractions before and after cellulose sulfate chromatography. 1:20 of input and eluted fraction were loaded on the gel.

The protein content of purified BVDV particles was analyzed by SDS-PAGE and colloidal Coomassie blue staining. As a control, we analyzed the protein content of an equivalent fraction originating from non-infected MDBK cells. An intense band migrating faster than the 17-kDa marker was present in the infected sample but not in the control (Fig 5A). The migration of this band was similar to the one detected by immunoblot with an anti-core antibody (Fig 5B) and to previous reports of BVDV core size [25]. The band of core appeared in less than a minute after addition of the staining solution, whereas other bands began to appear after several hours of staining, indicating that they were present in much lower amounts than core in the sample. Core was the only protein detected by Ponceau red staining, confirming its being the major protein present in the fraction (Fig 5B). The other bands detected in colloidal Coomassie blue staining were far less intense and were also detected in similar amounts in the non-infected control sample, suggesting that they are components of co-purified contaminating vesicles. A smeared band was detected in the infected sample, but not in the control, with a migration similar to that of E^rns^ monomer. However, surprisingly, no band could be clearly assigned to E1 or E2, when comparing virus and control samples, even though they were detected by immunoblot (Fig 5B), suggesting that BVDV particles have low amounts of envelope glycoproteins, or that BVDV envelope glycoproteins were not efficiently stained with colloidal Coomassie blue.

To further characterize the protein content of purified BVDV particles, proteins were analyzed by mass spectrometry. Proteins that were found at least twice out of 4 independent experiments, including one with proteins deglycosylated by PNGase F, are mentioned in Table 1. Two viral proteins, core and E^rns^, were identified, corresponding to the bands shown in Fig 5A, but E1 and E2 were not detected using Mascot searches. The other proteins identified were cellular proteins. They corresponded to proteins detected both in purified virus samples and in control fractions by Coomassie blue staining, and are likely components of contaminating vesicles detected in cryo-electron microscopy experiments.

**Table 1:** Proteins identified by mass spectrometry in purified BVDV fractions.

Protein	Origin	Gene ID	Nb of peptides matching	MS score	MSMS score/Nb of sequenced peptides
CD9P1	bovine	538209	17	76	nd
ALIX	bovine	533406	15	135	150/6 peptides
lactadherin	bovine	281913	14	185	51/4 peptides
E^rns^	BVDV	-	7	-	107/2 peptides
Actin	bovine	280979	11	107	128/4 peptides
Annexin A2	bovine	282689	11	127	125/5 peptides
CD9	bovine	280746	5	nd	116/4 peptides
Core	BVDV	-	6	-	305/6 peptides

To further assess the presence of E1 and E2 on purified BVDV, we compared the theoretical tryptic digest peak list of E1 and E2 with the experimental peak lists of samples corresponding to masses close to 25 or 55 kDa, respectively. Three masses corresponding to E2-derived peptides were found in a sample of ~55 kDa containing E^rns^ and lactadherin (S9 Fig). Due to the low intensity of these three peaks no MSMS experiment was carried out. These data strongly suggest the presence of E2, but do not strictly prove it. We performed a similar analysis for E1, but could not identify it. E1 runs as a ~25 kDa protein at a position very close to that of CD9, which is a major contaminant. We hypothesize that the presence of large amounts of CD9-derived peptides could have interfered with the detection of E1-derived peptides present in low amounts. The absence of detectable bands for E1 and E2 in Coomassie-stained gels and the lack of detection of E1 in mass spectrometry experiments further suggest that E1E2 content of BVDV envelope is low.

In contrast to HCV, no apolipoprotein was found associated to purified BVDV during the mass spectrometry experiments. Among the proteins detected, major cytosolic proteins, such as actin, tetraspanins network-associated proteins, such as CD9 and CD9P1, ESCRT proteins, such as ALIX, and phospholipid-binding proteins, such as lactadherin and annexin A2, have been previously found in purified exosomes from other cell types [27,28]. This suggests that contaminants found in purified BVDV fractions are exosomes. To assess whether some of these host factors could be virion components, we compared their distribution in a sucrose density gradient with the distribution of E2. The bands were quantified and plotted over fraction density. This revealed the presence of at least two populations of contaminating vesicles in addition to viral particles, one enriched in annexin A2, and the other one containing CD9 and ALIX (Fig 5C). CD9P1 and lactadherin could not be probed due to the lack of antibodies recognizing bovine proteins. The buoyant density of annexin-A2 peaked at 1.108 g/cm^3^ while virion marker E2 peaked at 1.127 g/cm^3^. The peak of the second population of contaminating vesicles was shared by fractions with densities of 1.127 g/cm^3^ and 1.136 g/cm^3^. Despite their distinct distributions, CD9, ALIX and, to some extent, annexin A2 overlapped E2 in the gradient (Fig 5C).

We also assessed whether some of these host factors would preferentially co-purify with the viral particles during the chromatography step. Cellulose-sulfate beads mimic heparan sulfate and preferentially bind viral particles. Therefore, host factors should co-purify with virions, if they are inserted in viral particles and should not bind beads if they are part of contaminating vesicles. Annexin A2 was detected in fractions of a chromatography experiment. Most annexin A2 did not bind cellulose-sulfate beads and was recovered in the flow-through fraction (Fig 5D). A minor amount of annexin A2 was detected in the 0.4 M NaCl virus elution fraction, where core was detected. A similar analysis was performed for ALIX, and CD9 (Fig 5E). Again most of the cellular proteins did not bind cellulose-sulfate beads, and a minor fraction was recovered in the virus fraction. All these results strongly suggest that most of these host proteins are part of contaminating vesicles, but we cannot completely rule out the possibility that a minor fraction of some of these proteins could be associated with viral particles.

### Lipid composition of purified BVDV particles

To determine if the low buoyant density of BVDV particles reflects an atypical lipid composition, as it was recently shown for hepatitis C virus particles [6], we quantified lipids of purified viruses. In parallel, we also quantified the lipid composition of infected and non-infected MDBK cells, in order to reveal any potential changes of lipid metabolism induced by BVDV infection, and to estimate the enrichment or reduction of lipids in the virions. Lipids were extracted from samples in the presence of internal lipid standards and subjected to nano-electrospray ionization MS/MS analyses on a QTRAP5500. Lipid species detection was performed in either precursor on neutral loss scanning mode, selecting for lipid class specific fragment ions that were generated by collision-induced dissociation under high vacuum. Data evaluation and quantitation of lipid species was done using the LipidView software. Twenty lipid classes (13 glycerophospholipids [GPLs], 3 sphingolipids [SLs], 3 neutral lipids and cholesterol) were included in the analysis. The GPLs analyzed were diacyl-phosphatidylcholine (PC), 1-alkyl,2-acylglycerophosphocholine or 1-acyl,2-alkylglycerophosphocholine (ePC), phosphatidylethanolamine (PE), 1-alkyl,2-acylglycerophosphoethanolamine or 1-acyl,2-alkylglycerophosphoethanolamine (ePE), 1-alkenyl,2-acylglycerophosphoethanolamine (also referred to as ethanolamine plasmalogen (pl-PE)), phosphatidylserine (PS), 1-alkyl,2-acylglycerophosphoserine or 1-acyl,2-alkylglycerophosphoserine (ePS), phosphatidylglycerol (PG, the mass of which is indistinguishable from its structural isomer lyso-bisphosphatidic acid, or LBPA), 1-alkyl,2-acylglycerophosphoglycerol or 1-acyl,2-alkylglycerophosphoglycerol (ePG), phosphatidylinositol (PI), 1-alkyl,2-acylglycerophosphoinositol or 1-acyl,2-alkylglycerophosphoinositol (ePI), phosphatidic acid (PA) and 1-alkyl,2-acylglycerophosphate or 1-acyl,2-alkylglycerophosphate (ePA). The three SLs analyzed were ceramide (Cer), sphingomyelin (SM), and hexosyl-ceramide (HexCer). The three neutral lipids were cholesteryl ester (CE), triacylglycerol (TAG) and diacylglycerol (DAG). Among the 20 lipid classes analyzed, a total of 398 different molecular lipid species were quantified (S1 Table).

**Fig 6:**
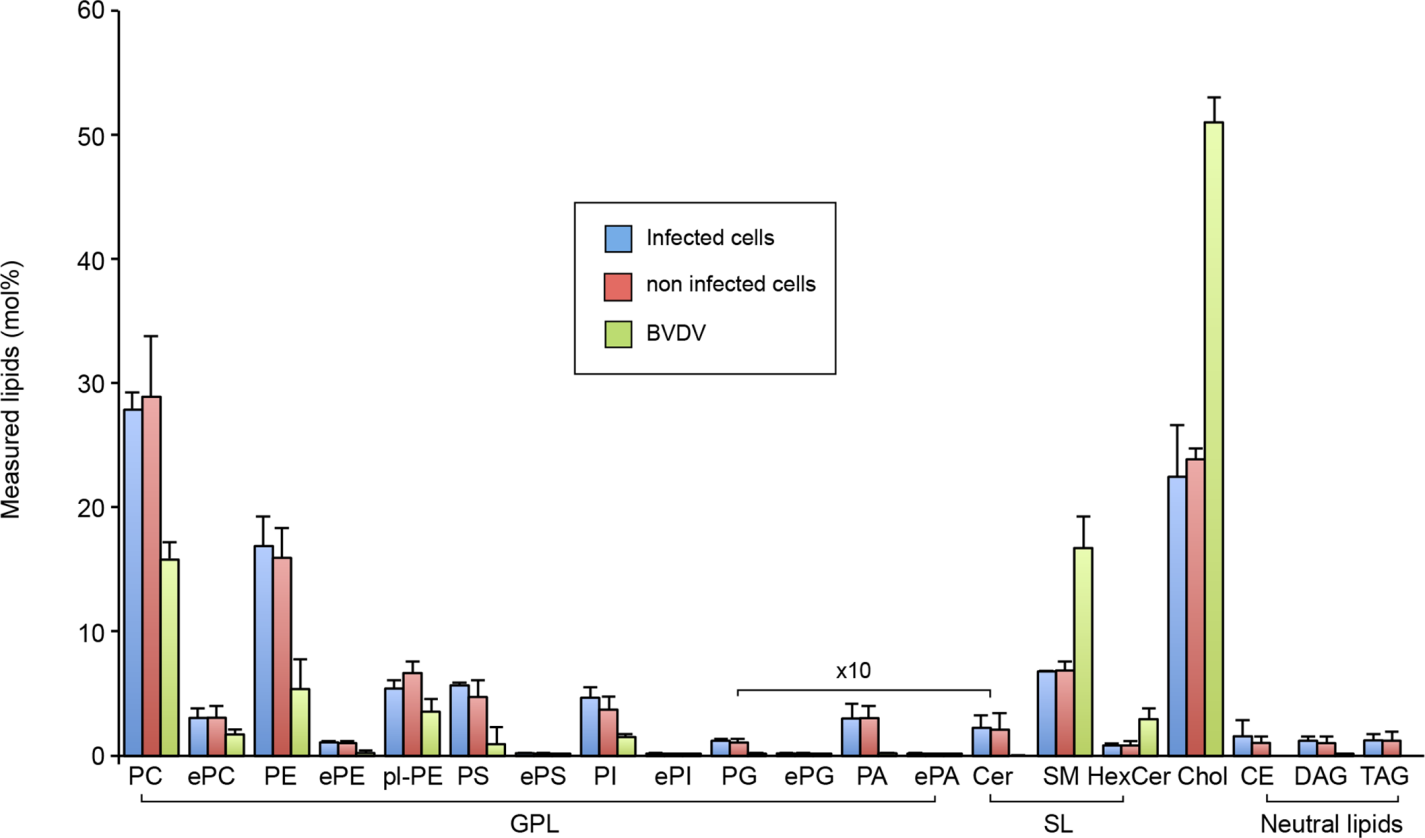
Lipid class composition of BVDV and MDBK cells. The content of individual lipid classes of infected MDBK cells, non-infected MDBK cells and purified BVDV were standardized to mole percentage of all membrane lipids within the sample. Amounts of lipids measured in control preparation from uninfected cells were subtracted from BVDV lipid values. Glycerophospholipids (GPL): phosphatidic acid (PA), phosphatidylcholine/-ethanolamine/-glycerol/-inositol/-serine (PC/PE/PG/PI/PS), ether linked GPL (ePC/ePE/ePG/ePI/ePS/ePA), and ethanolamine plasmalogen (pl-PE). Sphingolipids (SL): ceramide (Cer), sphingomyelin (SM) and hexosylceramide (HexCer). Sterols: cholesterol (Chol). Storage neutral lipids: cholesteryl esters (CE), diacylglycerol (DAG) and triacylglycerol (TAG). Error bars correspond to SDs (n = 3).

The lipidomes of BVDV particles and of total cellular membranes revealed a ~2.3 to 3.5 fold enrichment of cholesterol, SM and HexCer in the viral envelope as compared to cellular membranes (Table 2 and Fig 6). Cholesterol and SM account for 51 and 17 mol% of BVDV lipids, respectively. Together with HexCer, they contribute to more than 70 mol% of envelope lipids, while their abundance in MDBK cellular membranes is close to 30 mol%. This enrichment in cholesterol and SLs is mirrored by a 1.5 to 5 fold reduction of all GPL classes. In contrast, the lipid composition of infected and non-infected MDBK cells did not reveal any significant differences. Storage lipids CE, DAG and TAG were found in similar amounts in purified virus and in a control fraction obtained from non-infected cells. Therefore, they most likely represent components of contaminating material (S10 Fig). The same conclusion applies to Cer and PG, which were also measured in similar amounts in purified virus and control fractions.

**Table 2:** Lipid class composition of infected and non-infected MDBK cells and BVDV produced in MDBK cells.

Lipid	Avg mol% ± SD*		
	MDBK cells		BVDV**
	Infected	Non-infected	
PC	27.87 ± 1.45	28.92 ± 4.81	15.80 ± 1.35
ePC	3.06 ± 0.71	3.07 ± 0.92	1.73 ± 0.34
PS	5.69 ± 0.27	4.74 ± 1.26	0.95 ± 1.31
ePS	0.22 ± 0.02	0.17 ± 0.05	0.03 ± 0.01
PE	16.90 ± 2.44	15.95 ± 2.31	5.39 ± 2.47
ePE	1.09 ± 0.13	1.03 ± 0.14	0.23 ± 0.24
pl-PE	5.43 ± 0.64	6.68 ± 0.87	3,56 ± 1.06
PG	0.12 ± 0.02	0.11 ± 0.03	0.01 ± 0.01
ePG	0.02 ± 0.00	0.02 ± 0.00	NA***
PI	4.68 ± 0.85	3.73 ± 1.10	1.52 ± 0.33
ePI	0.19 ± 0.01	0.14 ± 0.01	0.01 ± 0.01
PA	0.30 ± 0.12	0.31 ± 0.09	0.02 ± 0.00
ePA	0.01 ± 0.01	0.01 ± 0.01	NA
Cer	0.23 ± 0.10	0.21 ± 0.14	NA
SM	6.81 ± 0.12	6.88 ± 0.68	16.74 ± 2.54
HexCer	0.85 ± 0.24	0.84 ± 0.37	2.96 ± 0.81
Chol	22.47 ± 4.24	23.88 ± 0.93	51.02 ± 1.99
CE	1.59 ± 1.24	1.05 ± 0.61	NA
DAG	1.22 ± 0.38	1.03 ± 0.54	0.02± 0.04
TAG	1.25 ± 0.53	1.23 ± 0.67	NA

*3 independent experiments

** lipids measured in non-infected control preparations were subtracted

***NA, not available (same amounts in control and BVDV preparations)

**Fig 7:**
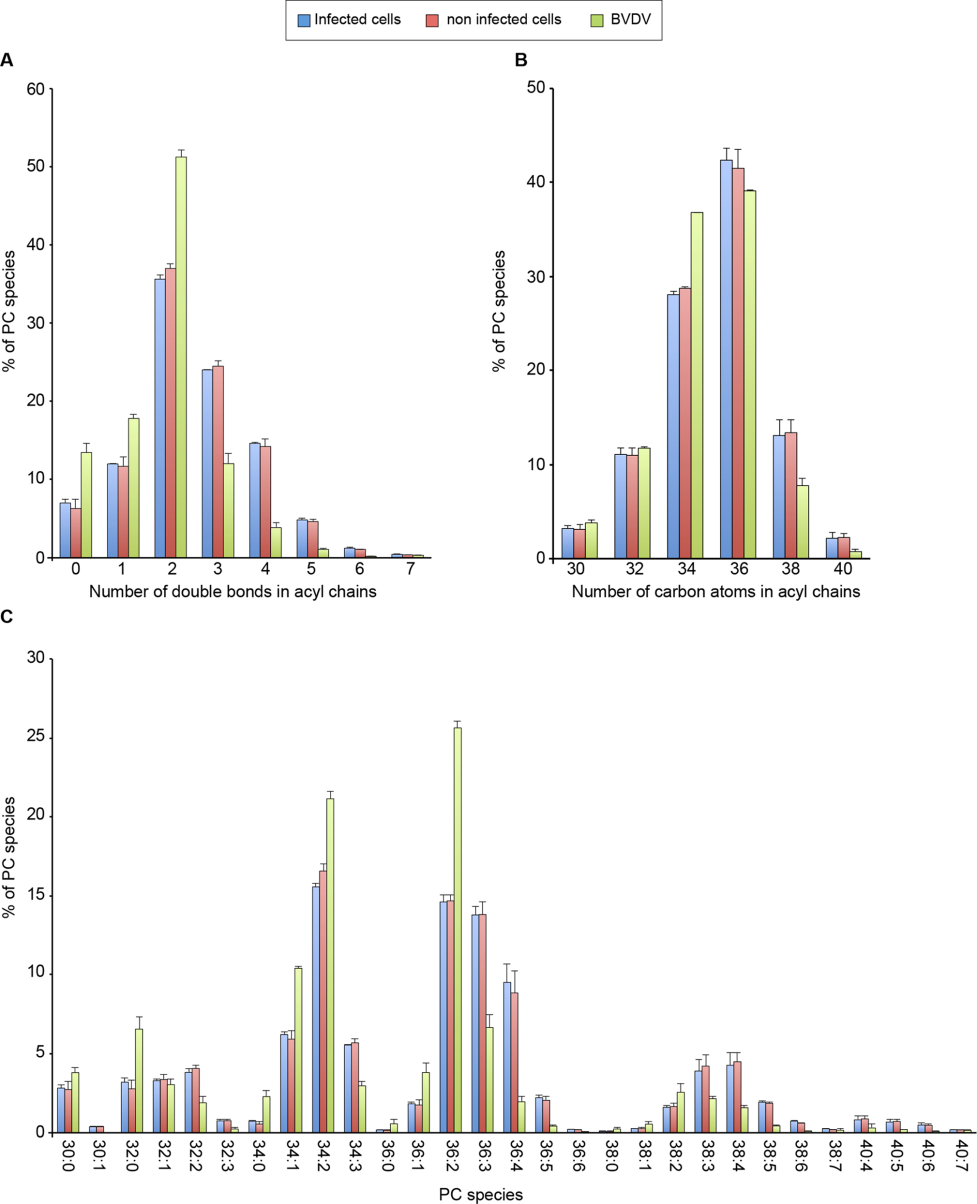
Phosphatidylcholine species of BVDV and MDBK cells. (A) PC saturation as a number of double bonds in both fatty acyl chains. (B) PC chain length as number of carbon atoms in both fatty acyl chains. (C) Molecular PC species. Amounts of each lipid were standardized to the total identified PC species. Error bars correspond to SDs (n = 3).

In addition to the differences observed for lipid classes, some differences could also be observed between individual lipid species of BVDV envelope and MDBK membranes. There is a tendency toward more saturated PC species in BVDV samples as compared to MDBK cells. PC species containing 0, 1 or 2 double bonds in acyl chains make up 82.5% of all PC species of BVDV and about 55% in MDBK cells (Fig 7A). Additionally, there is a shift toward shorter acyl chains. PCs with a sum of 34 carbon atoms in acyl chains were relatively more abundant in BVDV than in MDBK cells, at the expense of species with a sum of 36 carbon atoms or more in acyl chains (Fig 7B). As a result, PC 34:2 and PC 36:2, the major PC species represent 46.8% of all PC species in BVDV and 30.2% or 31.3% in infected and non-infected MDBK cells, respectively (Fig 7C). The tendency towards less unsaturated acyl chains is conserved, although less pronounced in PE and PI but not in other major GPL classes or in SLs (S11 Fig).

**Fig 8:**
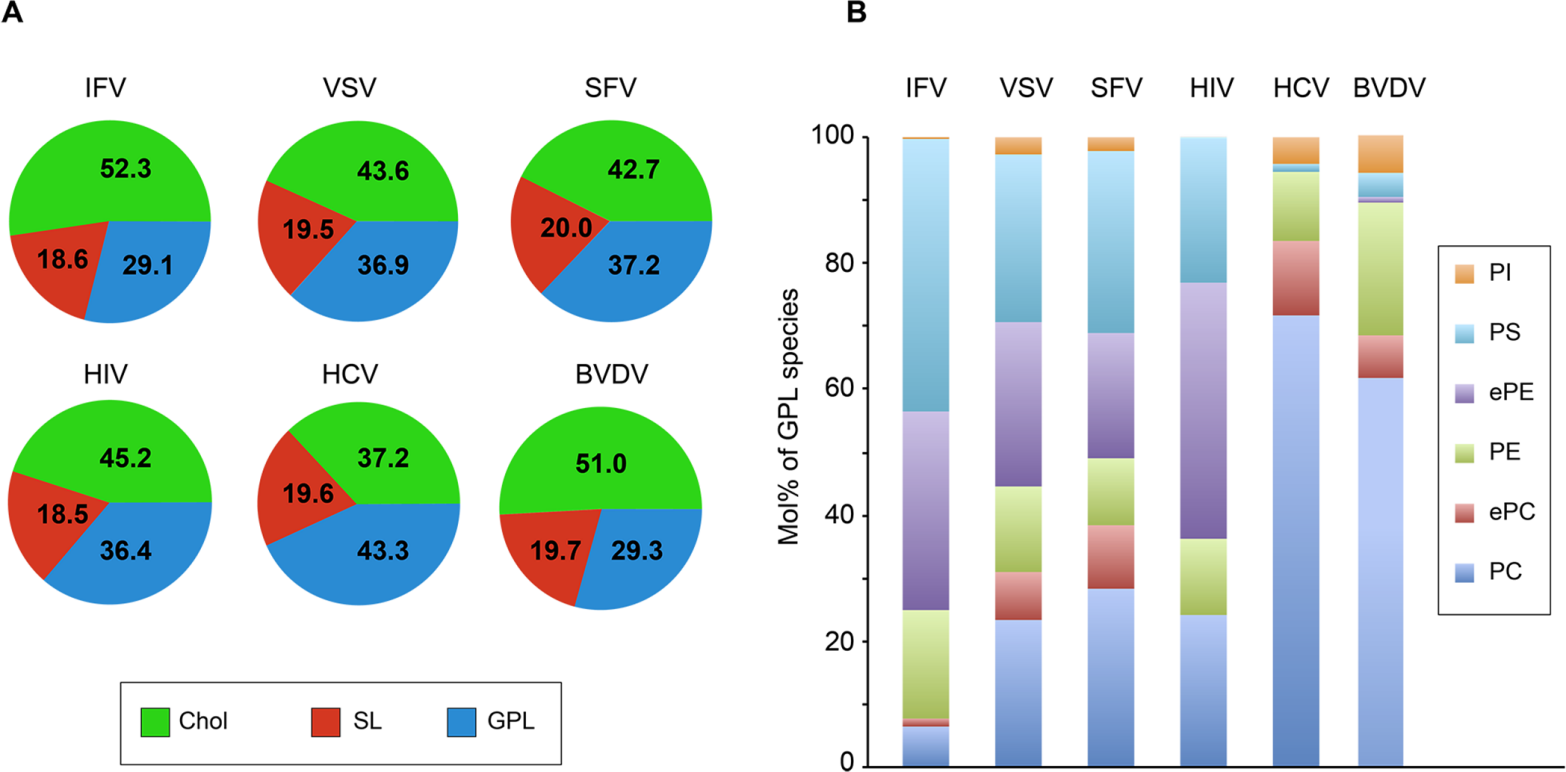
Lipid compositions of viruses. (A) Major lipid categories and (B) glycerophospholipid class compositions of influenza virus (IFV, ref #30), vesicular stomatitis virus (VSV), Semliki forest virus (SFV) (both taken from ref #29), human immunodeficiency virus (HIV, ref #28), hepatitis C virus (HCV, ref #6) and BVDV (this study). GPL: glycerophospholipid, SL: sphingolipid, chol: cholesterol, PI: phosphatidylinositol, PS phosphatidylserine, PE: phosphatidylethanolamine, PC: phosphatidylcholine, ePE: ether-linked PE, ePC: ether-linked PC. Cholesteryl esters of HCV have been omitted to facilitate the comparison with other viruses.

We also compared BVDV lipid content with other viruses [6,29–31]. Strikingly, BVDV was most similar to influenza virus, when the repartition of major lipid families such as cholesterol, SLs and GPLs, are compared. The distribution of major lipid families of its envelope was very different from that of HCV, another member of the *Flaviviridae* family, essentially because CE is the major lipid found in HCV [6] and is most probably absent from BVDV. Even when the storage lipid CE was omitted, BVDV and HCV distributions were still dissimilar, BVDV (51.0% Chol, 29.3% GPLs) being much closer to influenza (52.3% Chol, 29.1% GPLs) [31] than to HCV (37.2% Chol, 43.3% GPLs) [6], VSV (43.6% Chol, 36.9% GPLs), SFV (42.7% Chol, 37.2% GPLs) [30] or HIV (45.2% Chol, 36.4% GPLs) [29] (Fig 8A). All viruses had similar SLs content (18.5 to 20%).

Remarkably, when GPLs distributions were compared, BVDV and influenza were very different from each other. The major GPL of BVDV was PC (61.6% of all major GPLs), which is the main GPL found in the ER membrane [32]. In contrast, PC was detected in very small amounts (6.4%) in influenza virus [31]. Influenza virus envelope was enriched in ePE and PS (31.3 and 43%, respectively), while they represent only 0.9% and 3.8% of BVDV envelope GPLs (Fig 8B). In this respect, BVDV GPL content is closer, although clearly different, to that of HCV [6], another virus probably budding at the ER membrane, than to any other virus included in this analysis.

### Importance of sphingomyelin and cholesterol in BVDV infection

**Fig 9:**
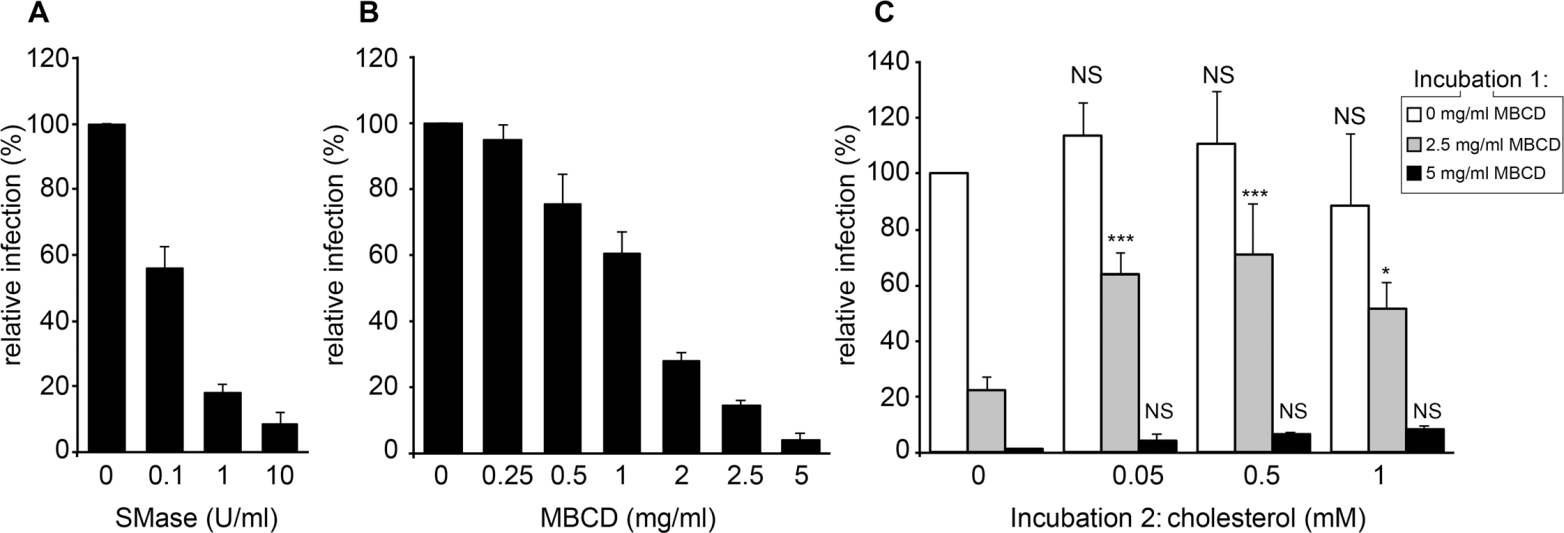
Sphingomyelin and cholesterol in BVDV entry. (A) Unpurified BVDV was incubated for 1h at 37°C in the presence of indicated concentrations of sphingomyelinase (SMase), diluted 1,000 times and used to infect MDBK cells. (B) Partially purified BVDV was incubated at 37°C for 1 h with indicated concentrations of methyl-β-cyclodextrin (MBCD), diluted 10,000 times and used to infect MDBK cells. (C) Partially purified BVDV was incubated at 37°C for 1 h with indicated concentrations of MBCD (incubation 1), diluted 100 times, incubated for 1h at 37°C with MBCD:cholesterol complex at final cholesterol concentrations indicated (incubation 2), diluted 100 times and used to infect MDBK cells. The number of infected cells was measured using an immunofluorescence assay at 15 hpi and standardized to the number of cells infected with untreated virus. Error bars correspond to SDs (n = 3, * P<0.05, *** P<0.001, NS: P>0.05; cholesterol-treated vs corresponding control, 2-way ANOVA).

To assess the functional importance of sphingomyelin and cholesterol, the two major lipids enriched in BVDV virions, we made use of sphingomyelinase and methyl-β-cyclodextrin, respectively. BVDV was pre-incubated with increasing amounts of sphingomyelinase (up to 10 U/ml), and then diluted in culture medium before infection of MDBK cells. A dose-dependent inhibition of BVDV infection was observed (Fig 9A). To ensure that this inhibition did not result from an action of sphingomyelinase on MDBK cells, a control experiment was performed with no pre-incubation with the virus before dilution and infection, and no inhibition of BVDV infection was detected (panel A in S12 Fig). Alternatively, cells were pre-treated with diluted sphingomyelinase in the absence of virus and then infected in the absence of sphingomyelinase, or infected in the absence of sphingomyelinase and then treated with diluted sphingomyelinase post infection. Again no impact on BVDV infection was measured with these conditions (panel A in S12 Fig). These results indicate that lipid modifications induced in BVDV envelope by sphingomyelinase treatment impaired BVDV infection.

BVDV was also treated with increasing concentrations of methyl-β-cyclodextrin, in order to extract cholesterol from its envelope, and then diluted before infection of MDBK cells. Again, a dose-dependent inhibition of BVDV infection was observed (Fig 9B). Control experiments with no pre-incubation did not show any inhibition of BVDV infection (panel B in S12 Fig), indicating an action of methyl-β-cyclodextrin on viral particles and not on cells. Importantly, when BVDV treated with 2.5 mg/ml methyl-β-cyclodextrin was further incubated with cholesterol-loaded methyl-β-cyclodextrin before infection of MDBK cells, in order to replenish the cholesterol content of the viral envelope, the infectivity was partially restored (Fig 9C). In contrast, BVDV treated with 5 mg/ml methyl-β-cyclodextrin could not be rescued by cholesterol addition. This suggests that a strong cholesterol extraction from the viral envelope irreversibly inactivates the virus, whereas a partial cholesterol extraction impairs BVDV infection but does not irreversibly disrupt the viral envelope.

Examination of BVDV by cryo-EM confirmed the integrity of virions in samples treated with 1 U/ml sphingomyelinase or 2.5 mg/ml methyl-β-cyclodextrin (S13 Fig). This indicates that the loss of infectivity is not due to the viral particles being destroyed and indeed results from the depletion of specific lipids from the envelope. In contrast, the sample treated with 5 mg/ml methyl-β-cyclodextrin, which did not allow infection rescue by cholesterol replenishment (Fig 9C), contained no virus (S13 Fig). Only a few non-viral intact vesicles, probably derived from cholesterol-poor contaminating membranes, and a number of large aggregates containing membranous material were observed (S14 Fig). This observation explains the lack of cholesterol-mediated rescue with this dose of methyl-β-cyclodextrin. These results confirmed the functional importance of sphingomyelin and cholesterol in BVDV entry.

### Assessment of the role of lipid rafts for BVDV assembly

**Fig 10:**
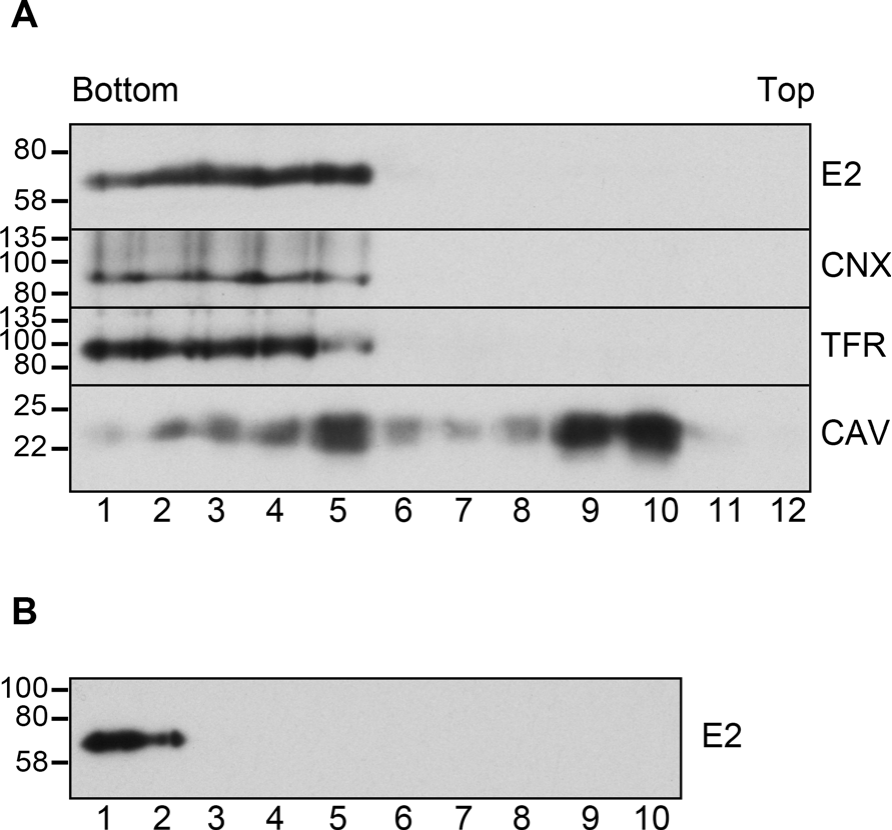
E2 is excluded from detergent-resistant membranes. DRMs were isolated by cold extraction of BVDV-infected MDBK cells (A) or partially purified BVDV (B) with Triton-X100 and flotation as explained in the Materials and Methods section. The distribution of BVDV E2, calnexin(CNX), transferrin receptor (TFR) and caveolin (CAV) was determined by immunoblot under reducing (CNX, TFR) or non-reducing (E2, CAV) conditions.

The lipid composition of BVDV envelope suggests that it might bud in membrane domains enriched in cholesterol and SM. Membranes enriched in cholesterol and sphingolipids have a tendency to produce lipid raft domains [33]. These structures are thought to be insoluble and coalesce in cold non-ionic detergents and can be isolated by flotation in a sucrose density gradient as proteo-lipidic complexes named detergent-resistant membranes, or DRMs [34]. To determine if BVDV envelope glycoproteins can be found in DRMs, BVDV-infected cells were lysed on ice in TNE containing Triton X-100 and the lysate was submitted to flotation in a sucrose density gradient in order to resolve DRMs from soluble proteins. Most of caveolin (a DRM marker) was found in floated fractions, whereas the transferrin receptor (a protein excluded from DRMs) was not (Fig 10A). We also probed the distribution of calnexin, a trans-membrane protein of the ER, and did not observe any signal in floated fractions, as expected, indicating that the ER membrane was well solubilized. Under these experimental conditions, E2 was not found in DRM fractions from infected cells lysates (Fig 10A) and in floated fractions from BVDV particles lysates (Fig 10B). This indicates that, despite a high cholesterol and SM content of BVDV envelope, E2 does not appear to be included in raft-like structures of the ER membrane or the viral envelope.

## Discussion

### Purification of BVDV particles

In this study, we report on the first purification of BVDV particles and the description of their morphology and their molecular composition. Due to moderate viral titers and to the presence of contaminating vesicular material of cellular origin, the four-step purification we set up yields a virus ~90% pure (Fig 1 and Fig 2). The contaminating material included two distinct vesicle populations with different buoyant densities and protein content (Fig 5). One of these vesicle populations with a density of about 1.14 g/cm^3^ and containing exosome markers such as the tetraspanin CD9 and the ESCRT protein ALIX can be identified as exosomes originating from intraluminal vesicles of multivesicular bodies [27,28]. The other population, which is lighter and contains annexin A2, might have another cellular origin. Importantly, both of these cell-derived vesicle populations are also present in conditioned medium of non-infected MDBK cells, clearly indicating that they are unrelated to BVDV infection and viral morphogenesis. Nevertheless, the presence of limited amounts of contaminating vesicles did not impede morphological and biochemical studies of BVDV particles. One key issue to achieve this purification was the use of a non-cytopathic strain in order to minimize the amounts of contaminating material in supernatants of infected cells. For this reason, attempts to purify BVDV particles from the cytopathic strain NADL were unsuccessful (S6 Fig), despite higher titers.

### Morphology of BVDV particles

Purified BVDV virions appeared in electron microscopy as spherical particles with a diameter of 50 nm (Fig 2), in keeping with previous observations of cell-associated BVDV particles [16,23]. In cryo-electron microscopy, virions are characterized by an electron-dense core surrounded by a lipid bilayer, which displays a smooth surface with no spikes (Fig 2 and S1 Fig). This could reflect the presence of envelope glycoproteins lying parallel to the lipid bilayer, like E dimers of flavivirus mature virions [3,35]. However, acidic treatment of purified BVDV virions did not induce the formation of spikes (S7 Fig), in contrast to what has been reported for flaviviruses [36]. This difference of response to an acidic treatment may result from the pH-insensitivity of BVDV [22], or the structural organization of BVDV envelope glycoprotein E2, which is very different from class II glycoprotein E of flaviviruses [10,11]. Alternatively, this may also reflect the presence of low amounts of envelope glycoproteins at the surface of BVDV particles, a hypothesis that is also supported by biochemical data (Fig 5 and S9 Fig).

Interestingly, the vast majority of viral particles observed in cryo-electron microscopy have a uniform size of approximately 50 nm, except for a low number (about 2%) that are larger with a diameter up to 65 nm (Fig 2 and S1 Fig), which corresponds to a volume twice as large. We cannot exclude that these larger particles could be aberrant forms related to cell culture conditions. However, BVDV is known to be able to incorporate pieces of host RNA into its genome [14]. We can speculate that a size flexibility of the virion would be an advantage for the virus to accommodate larger genomes. To our knowledge, such a size flexibility of the virion has not been observed for flaviviruses. This suggests different structural organization of the capsid of pestiviruses and flaviviruses. The polymorphic nature of a viral particle may arise from non-icosahedral structure, as in rubella virus [37], or from variations in quasi-symmetry or triangulation number of icosahedral structure, as in hepatitis B virus capsid [38]. Whether BVDV capsid structure is isosahedral or not must await studies with higher resolution cryo-EM techniques.

The purified virus preparation also contained non-enveloped particles corresponding to putative naked capsids (Fig 2 and S1 Fig). This is most likely an artifact of the last step of purification, for two reasons: non-lipidated particles, if infected cells were to secrete them, should have a buoyant density quite different from that of enveloped particles and be discarded during the two steps of ultracentrifugation. However we always detected a single peak of capsid-containing material in these two ultracentrifugation steps. Moreover, cryo-electron microscopy examination of virus fractions obtained after the flotation gradient but without chromatography on sepharose-sulfate beads did not reveal any naked capsid-like particle. This strongly suggests that non-enveloped capsids are generated during the last step of purification.

### Protein content of BVDV particles

One surprising finding of this study is the apparent low glycoprotein content of BVDV particles. E1 and E2 were never detected in colloidal Coomassie blue-stained gels, while core was very abundant and even some proteins of the ~10% contaminating material were easily detected (Fig 5). On the other hand, E^rns^ was detected as a smear, however apparently in lower amounts than core (Fig 5). This suggests that BVDV particles have a higher content of E^rns^ than E1 and E2, a conclusion that is also supported by results of mass spectrometry analysis. E1 was not detected in four independent experiments, and E2 could be identified only in low amounts (S9 Fig), while E^rns^ could easily be identified. The deglycosylation of viral particles with PNGase did not improve E1 and E2 detection in mass spectrometry (Table 1), suggesting that their low levels of detection did not simply result from hindrance caused by their carbohydrate moieties. These lower amounts of E1 and E2 were previously suggested from immunogold labeling of BVDV particles in electron microscopy [15]. Lower levels of E1E2 are surprising because E1 and E2 are required for pestivirus entry and E^rns^ is not [39,40]. However, we should keep in mind that it has been reported for other viruses that a very low number of functional envelope proteins may be required for entry [41–44].

E1 and E2 could be detected in purified BVDV particles by immunoblotting (Fig 1, Fig 4 and Fig 5), by neutralization (S4 Fig) and by immunocapture (Fig 3). As reported earlier [23,45], E1 and E2 were found as covalent heterodimers. In contrast, cell lysates of infected cells showed a mixture of monomers, covalent heterodimers and covalent E2 homodimers (Fig 4). This suggests that a mechanism operates during morphogenesis that allows covalent heterodimers to be specifically incorporated in the nascent particle. Alternatively, E1 and E2 disulfide bridges could be reshuffled during assembly. The presence of covalent heterodimers is consistent with the report that only covalent E1E2 heterodimers are required for BVDV entry, and that covalent E2 homodimers are inactive [40]. In previous studies, E2 covalent homodimers were detected in unpurified pestivirus particles in addition to covalent E1E2 heterodimers [23–25]. It is not yet clear whether the presence of E2 homodimers in virions is strain-specific, or if it merely reflects the presence of contaminating vesicles of cellular origin. For example, material secreted by MDBK cells infected by cytopathic strain NADL contained both homodimers and heterodimers. Although we did not succeed in purifying NADL particles, we observed that the homodimer:heterodimer ratio varied during purification (S8 Fig), suggesting that they are present in different populations of vesicles in the starting material.

### Lipid content of BVDV particles

A major finding of this study is the unexpected lipid composition of BVDV envelope (Fig 6, Fig 7 and Table 2). The lipid composition of the viral envelope does not match any known intracellular membrane. Its cholesterol and SM content are somewhat similar to what could be found in the plasma membrane of some cells, and in envelopes of viruses budding at the cell surface (Fig 8). However PS and ethanolamine plasmalogens, two phospholipids enriched in the plasma membrane and viruses budding at the plasma membrane [29–31,46] were found in low amounts in BVDV envelope, suggesting that the lipid bilayer of the viral envelope is not likely to derive from the plasma membrane. Similarly, the very low ceramide and PG/LBPA content of BVDV envelope does not support a budding event in multivesicular endosomes. A high content of cholesterol and sphingolipids is often associated with lipid rafts [33]. However, ethanolamine plasmalogens, another marker of lipid rafts [47], are not enriched in BVDV envelope (Fig 6 and Table 2). Moreover, viral glycoprotein E2 was not found in DRMs of infected cells or purified virus (Fig 10). These observations are not in favor of BVDV budding in lipid raft microdomains of MDBK cells.

Several lines of evidence are in favor of BVDV budding into the ER. Envelope glycoproteins are located at the ER membrane of infected cells [48,49]. Infectious BVDV particles accumulate intracellularly upon brefeldin A treatment [50]. Individual budding events at the ER membrane have been observed by electron microscopy [16]. In contrast, the BVDV envelope lipid composition, and especially its cholesterol and SM content, is quite different from a typical ER membrane [32,51], even though the lipid content of the ER of MDBK cells has not been determined to our knowledge. Interestingly, SM has been recently shown to be involved in the morphogenesis of West Nile virus [52], a flavivirus that also buds in the ER, suggesting a potential role of SM in viral budding in the ER. On the other hand, if one only considers GPLs, it is worth noting that BVDV envelope is quite similar to ER membrane [32,51] and to the envelope of HCV, another virus budding in the ER [6], which both contain high amounts of PC and PE (Fig 8). These data suggest that the distribution of GPLs could be more representative of the membrane where the virus buds than the full lipid repertoire.

Remarkably, BVDV and influenza virus, two viruses budding from cellular membranes so different as ER and apical PM, reach similar proportions of GPLs, SLs and cholesterol (Fig 8). Their high cholesterol and SLs contents are likely to render their envelopes very sturdy and help them to resist to harsh conditions found outside their hosts. It is tempting to hypothesize that this is an example of convergent evolution driven by similar modes of transmission. Influenza lipid content is very similar to apical PM content and the formation of its envelope only involves minor sorting events of selected lipid species [31]. On the other hand, BVDV envelope lipid content is highly divergent from a typical ER membrane composition. Three different hypotheses could explain this observation. A first hypothesis is that the viral infection could alter the cellular metabolism in such a way as to modify the lipid composition of the cellular membrane where the virus buds, here the ER membrane. However this hypothesis is unlikely, because we did not detect any difference of lipid content between infected and non-infected cells (Table 2). Actually, BVDV replication has been associated to very discrete membrane rearrangements [53]. This absence of any major membrane rearrangement is in line with the absence of lipid content modification during BVDV infection. A second hypothesis is that BVDV would bud into microdomains of the ER membrane enriched in cholesterol and SM, whether they are normally present in the ER membrane or their formation is induced during BVDV infection. Although ER is known not to be enriched in SM and cholesterol, evidence for the presence of DRMs in the ER has been reported [54,55], suggesting a mosaic composition of the ER membrane. However, our results do not support BVDV budding in lipid rafts (Table 2 and Fig 10). Alternatively, a third hypothesis is that viral proteins could recruit/concentrate cholesterol and sphingolipids to nascent viral envelope during the budding process. It is unlikely that E2 could drive such a process, because of its low amounts in virions and its low affinity to membranes enriched in cholesterol and SLs. Viral proteins fitted for performing such a function might be core, E^rns^ and/or non-structural proteins involved in the assembly process, such as NS3 [17].

Our data add to the notion that members of different genera of the *Flaviviridae* family use different mechanisms for their morphogenesis. Unlike flaviviruses, HCV [6,7] and BVDV (this work) appear to contain a limited number of envelope glycoproteins per virion. BVDV also differs from HCV, which uses the machinery of VLDL formation of hepatocytes [4]. The absence of associated apolipoproteins and the very low amounts of CE and TAG, if any, in BVDV particles clearly indicates that BVDV does not rely on lipoproteins for its morphogenesis, in agreement with previous demonstration that LDL receptor plays no role during BVDV entry [56]. Our results further suggest that major changes of lipid content in the bilayer originating from the ER membrane are involved in the formation of BVDV envelope. Future studies on molecular and cellular mechanisms operating this process of lipid sorting should indicate important aspects of pestivirus morphogenesis.

## Materials and Methods

### Chemicals

Dulbecco’s modified Eagle’s medium (DMEM), phosphate buffered saline (PBS), glutamax, horse and goat sera, DAPI, gentamycine, nonessential amino acids and trypsin/EDTA were purchased from Life Technologies. All other chemicals were purchased from Sigma.

### Cell culture

BVDV-free MDBK cells (ATCC number CCL-22) were propagated in DMEM, high glucose modification, supplemented with 10% heat-inactivated horse serum, nonessential amino acids and 2 mM glutamax at 37°C in a humidified atmosphere containing 5% CO_2_, and were passaged by trypsination twice a week.

### Virus

BVDV genotype 1 non-cytopathic strain WAX-N used in this study was obtained from P.P. Pastoret (University of Liège, Belgium), and maintained by passages in MDBK cells. The coding sequence of the N-terminal part of the polyprotein (Npro-p7), which encompasses the structural proteins, was determined from RT-PCR fragments amplified from total RNA of infected cells using primers listed in table A in S1 Text, and deposited to Genebank (accession number KR013753).

BVDV cytopathic strain NADL was obtained from C. M. Rice (The Rockefeller University, New York).

### Virus titration

About 10^4^ MDBK cells were seeded in wells of P96 plates and infected 4 hours later with 10-fold dilution series of BVDV samples. The medium was replaced after 1 hour of contact. Cells were fixed at 18 to 20 hpi with 3% paraformaldehyde and processed for immunofluorescent detection of NS3. Immunofluorescent foci were counted from wells infected with appropriate BVDV dilution, and viral titers were expressed as focus-forming units (ffu) per ml.

### Antibodies

#### Anti-BVDV core polyclonal antibody

The core-coding sequence of BVDV strain NADL was PCR-amplified from pACNR-BVD NADL [57], subcloned into pGEX-4T1 and a GST-core fusion protein was produced in *E*. *coli* BL21 (DE3). Polyclonal anti-BVDV core antiserum was prepared by immunizing a rabbit with affinity-purified GST-core. Anti-core IgG were affinity-purified from immune serum. To this end, a His_6_-tagged version of BVDV core was expressed in *E*. *coli* BL21 (DE3), purified by affinity chromatography (Ni-NTA superflow cartridges, Qiagen), and coupled to CNBr-activated sepharose-4B. The IgG fraction of the immune serum was recovered by fractionated ammonium sulfate precipitation, and run through the His_6_-core-sepharose 4B affinity column. Bound IgG was eluted with 4 M MgCl_2_, 10 mM Na_2_PO_4_, pH 7.4 and dialyzed against PBS (PBS is 137 mM NaCl, 2.7 mM KCl, 1.2 mM KH_2_PO_4_, 8.1 mM Na_2_HPO_4_, pH 7.0).

#### Generation of BVDV E1 monoclonal antibody

In a first step, a PCR fragment containing murine ubiquitin (ubiN: TTTCATATGCAGATCTTCGTGAAG; ubiC: AAAGGATCCGCCACCGCGGAGTCGCAGCAC) was generated and cloned into the NdeI-BamHI sites of pET26b. Next, the ectodomain of E1 of BVDV NCP-7 was amplified with oligonucleotides BVTK94 (AAAGATCTGCCCTTCTCCCTATTGTGAGG) and BVTK121 (TCTCGAAGATCTGGTCTTACACAAAC) complementary to nt 1877–1892 and nt 2236–2254, respectively of BVDV NCP7) and cloned via EcoRI and XhoI between the ubiquitin gene and the hexa His tag, yielding plasmid pTK232. The ubiquitin-E1 6xHis protein was expressed in Rosetta DE3 cells at 30°C for 4h after induction with 1mM IPTG. The insoluble protein was solubilized using 6M guanidium hydrochloride (Sigma) and purified by chromatography on HisTrap columns (GE healthcare) [58]. 0.5mg of purified protein was desalted and served for immunization of 2 BalbC/black mice after adding Gerbu Adjuvant MM as recommended by the manufacturer (Gerbu, Heidelberg). Mice were immunized a total of 6 times before spleenocytes were fused with SP2/0 cells as described [58]. Hybridomas were screened using the ubi-E1 fusion protein or an unrelated ubiquitin fusion protein for differentiation by ELISA. A single hybridoma (8F2) was recovered that detected the E1 moiety in ELISA, Western blot and immunofluorescence.

#### Other antibodies

The mouse mAb OSC-23 to BVDV NS3 protein [59] was kindly provided by P.P. Pastoret (University of Liège, Belgium) and G. Chappuis (Merial company, France). MAb OSC-23 was used as unpurified culture medium from hybridoma grown in a miniperm apparatus (Heraeus). Rabbit polyclonal anti-E^rns^ was kindly provided by Gregor Meyers, Friedrich-Loeffler-Institut, Greifswald—Insel Riems, Germany. Mouse mAbs anti-BVDV E2 WB166 and WB214 were obtained from Veterinary Laboratories Agency, Addelstone, UK. Anti-bovine CD9 mouse mAb IVA50 was from Novus. Anti-annexin A2 mouse mAb was from Life Technologies. Anti-KDEL mouse mAb (used as irrelevant in immunocapture experiments) was from Stressgen. Anti-calnexin rabbit pAb was from Enzo Life Sciences. Anti-transferrin receptor mouse mAb H68.4 was from Santa Cruz Biotechnology. Anti-caveolin rabbit pAb was from Transduction Laboratories.

### Purification of BVDV particles

Approximately 3.10^8^ MDBK cells were infected in suspension with occasional mixing at an M.O.I. of approximately 0.02 for 1 hour at room temperature in a volume of 20 ml DMEM containing 10% horse serum and then diluted to 450 ml of DMEM containing 2% horse serum and plated in 3 cell factories (Easyfill-2trays, Nunc). Cell culture supernatants were collected at 40 hpi and then again 24 h later. Both cell culture supernatants were combined and centrifuged at 5000 rpm, 4°C for 15 minutes in a JS-7.500 rotor (Beckman) to get rid of cellular debris. Polyethyleneglycol (PEG)-6000 (7%) was slowly added to supernatants under stirring in order to allow rapid solubilization. The mixture was incubated at 4°C under slow continuous stirring for 4 hours and centrifuged at 10,000 rpm, 4°C for 30 minutes in a JLA-10.500 rotor (Beckman). The pellet was resuspended in 7 ml TNE (TNE is 20 mM TrisCl pH 8.0, 150 mM NaCl, 2 mM EDTA) and incubated overnight at 4°C. Insoluble material was removed by centrifugation (7000 rpm, 4°C for 10 minutes in a JS-7.500 rotor). The supernatant was loaded over 2 layers of 30% (2ml) and 15% (3 ml) sucrose (w:v) in TNE in a 12-ml ultraclear centrifuge tube (Beckman) and centrifuged at 35,000 rpm, 4°C for 2 hours in an SW 41 rotor (Beckman). Fractions (0.5 ml) were collected dropwise after puncturing the bottom of the tube. The fraction containing the major part of infectious virus and core protein was mixed with 1 volume of 60% sucrose in TNE, loaded under a 5–35% sucrose gradient, and centrifuged at 35,000 rpm, 4°C for 20 hours in an SW 41 rotor. Fractions (0.5 ml) were collected dropwise after puncturing the bottom of the tube. The distribution of the virus in the gradient was measured by titration and/or core immunoblotting. The density of each fraction was measured with a refractometer. One or two fractions of the gradient containing the peak of infectious virus were loaded on a 0.5-ml column of sulfated cellulose (Cellufine Sulfate, Chisso Corporation) pre-equilibrated with TNE. After virus binding, the column was washed with 3 volumes of PBS and viral particles were eluted in 1 ml of PBS containing 0.4 M NaCl. Eluted fraction was centrifuged at 45,000 rpm, 4°C for 3 hours in a TLA-55 rotor (Beckman) and the pellet of purified virus was resuspended in 50 μl PBS. For electron microscopy analysis, the virus was kept at 4°C in PBS until analysis. For lipid extraction and analysis, two preparations were pooled. The final pellet was resuspended in 155 mM ammonium bicarbonate and kept at -80°C.

### Cryo-electron microscopy

Firstly a continuous carbon film was deposited on the carbon face of a quantifoil with circular holes cupper grid (Ted Pella Inc). BVDV samples (5 μl) were deposited onto the cupper surface of the grid placed in the automated device for plunge-freezing (EM GP Leica) enabling a perfect control of temperature (15°C) and relative humidity (70% RH). The excess of sample was blotted with filter paper and the grid was plunged into a liquid ethane bath cooled and maintained at -183°C with liquid nitrogen. Specimens were maintained at a temperature of approximately -170°C, using a cryo holder (Gatan, CA, USA) and observed with a FEI Tecnai F20 electron microscope operating at 200 kV and at a nominal magnification of 29,000 x under low-dose conditions. Images were recorded with a 2k x 2k USC 1000 slow-scan CCD camera (Gatan). Pixel size was 0.37 nm.

### Immunocapture of BVDV particles using magnetic beads

Protein A and protein G-conjugated superparamagnetic beads (Bio-Adembeads) were purchased from Ademtech. Before use, the beads were washed with a large volume of working buffer (PBS). The magnetic beads (10 μl) were incubated with anti-BVDV E2 mAb or anti-KDEL mAb (control) in a final volume of 200 μl, at 4°C for 30 min. The magnetic bead–protein A/G–antibody complexes were sedimented with a magnet. The pellets were washed three times to remove excess antibody. Beads coated with antibody were incubated with BVDV at room temperature for 30 min (final volume 100 μl). After sedimentation with the magnet, the pellet was washed in order to remove unbound particles. Cryo-TEM lacey carbon cupper grids were then prepared with this suspension, without any further treatment according to the protocol described above. Specimens were maintained at a temperature of approximately -170°C, using a cryo holder (Gatan, CA, USA) and observed with a FEI Tecnai12 electron microscope operating at 120 kV and at a nominal magnification of 30,000 x under low-dose conditions. Images were recorded with a 4k x 4k slow-scan CCD camera (FEI).

### Protein identification by mass spectrometry

Proteins of purified virus or a corresponding fraction from non-infected cells were separated under reducing conditions in a 12% polyacrylamide gel. In one experiment, half of a purified virus preparation was denatured and deglycosylated overnight at 37°C with PNGase F (P0704, New England Biolab) as recommended by the manufacturer, before gel electrophoresis. The gel was stained for 3 days in a solution of colloidal Coomassie blue. Stained bands were excised from the gel, reduced, alkylated with iodoacetamide (10 mg/ml in NH_4_HCO_3_ 20 mM) and digested overnight with 50 ng trypsin (Promega) in 20 mM NH_4_HCO_3_. The resulting peptides mixtures were eluted from the gel, desalted, and spotted on a MALDI plate with freshly dissolved α-cyano-4-hydroxycinnaminic acid (10 mg/ml in 50% CH_3_CN, TFA 1/1000). Mass spectrometry was performed with a MALDI-TOF-TOF Autoflex Speed (Bruker Daltonics).

MS and MS/MS data were analyzed using BioTools software. Identification of peptides was performed using Mascot, http://www.matrixscience.com/.

### Lipid extraction and mass spectrometry

Lipid extractions were performed using chloroform:methanol:37% HCl (5:10:0.15, vol:vol:vol,) except for plasmalogens, which were extracted using chloroform/methanol (5:10, vol:vol) as extraction solvent. Lipid extractions were done in 10 ml Wheaton vial with Teflon-screw caps as described [60]. Typically, the following lipid standards were added to the solvent prior to extractions: 100 pmol of PC (13:0/13:0, 14:0/14:0, 20:0/20:0; 21:0/21:0, Avanti Polar Lipids), SM (d18:1 with N-acylated 15:0, 17:0, 25:0, semi-synthesized as described in [36]) and d6Chol (Cambrigde Isotope Laboratory), 55 pmol PI (16:0/16:0, 17:0/20:4, Avanti Polar Lipids), 50 pmol PE and PS (14:1/14:1, 20:1/20:1, 22:1/22:1, semi-synthesized as described in [60], DAG (17:0/17:0, Larodan) and cholesterol ester (CE, 9:0, 19:0, 24:1, Sigma), 40 pmol TAG (D5-TAG-Mix, LM-6000 / D5-TAG 17:0,17:1,17:1 –Avanti Polar Lipids), 10 pmol Cer and GlcCer (d18:1 with N-acylated 15:0, 17:0, 25:0, semi-synthesized as described [60], PA (PA 17:0/20:4, Avanti Polar Lipids) and PG (14:1/14:1, 20:1/20:1, 22:1/22:1, semi-synthesized as described in [60]. Neutral extraction solvents were spiked with 100 pmol PC standard mix and plasmalogen PE (pl-PE) standard mix containing 66 pmol pl-PE Mix 1 (16:0p/15:0, 16:0p/19:0, 16:0p/25:0), 93 pmol pl-PE Mix 2 (18:0p/15:0, 18:0p/19:0, 18:0p/25:0) and 129 pmol pl-PE Mix 3 (18:1p/15:0, 18:1p/19:0, 18:1p/25:0). Semi-synthesis of pl-PE was performed as described in [61]. For lipid analysis, 10–20 μl of purified virions and 1-5x10^4^ cells were subjected to extractions. Mass spectrometric analysis was performed on a QTRAP5500 (ABSciex) coupled to a Triversa NanoMate device (Advion) as described [60]. Data processing was performed using LipidView (ABSciex), Microsoft Excel and a custom-made data evaluation program (ShinyLipids).

### Immunoblotting

Cells were incubated in lysis buffer (50 mM Tris-Cl buffer pH 7.5 containing 100 mM NaCl, 1 mM EDTA, 1% Triton X-100, 0.1% sodium dodecyl sulfate (SDS), and protease inhibitors) for 20 min on ice. Nuclei were pelleted by centrifugation. The protein concentration in post-nuclear supernatants was determined by the bicinchoninic acid method as recommended by the manufacturer (Sigma), using bovine serum albumin as standard. Proteins from cell lysates or virus purification fractions were separated by SDS-polyacrylamide gel electrophoresis and transferred to nitrocellulose membranes (Hybond-ECL; Amersham) by using a Trans-Blot apparatus (Bio-Rad). The proteins of interest were revealed with specific primary antibodies, followed by species-specific secondary antibodies conjugated to horseradish peroxidase (Jackson Immunoresearch), and enhanced chemiluminescence detection as recommended by the manufacturer (Thermofischer).

### Sphingomyelinase and methyl-β cyclodextrin treatments

Unpurified BVDV was incubated in culture medium for 1 h at 37°C with sphingomyelinase from *Bacillus cereus* (Sigma S7651) at various concentrations. Treated virus was then diluted 1,000 times in culture medium before infecting MDBK cells.

Partially purified BVDV (15–30% sucrose interface) was incubated in culture medium for 1 h at 37°C in the presence of various concentrations of methyl-β-cyclodextrin. Treated virus was then either diluted 10,000 times in culture medium and used to infect MDBK cells, or diluted 100 times in DMEM containing various concentrations of cholesterol:methyl-β-cyclodextrin complex and incubated for 1 h at 37°C in order to reload methyl-β-cyclodextrin-treated virus with cholesterol. Treated virus was diluted 100 times before infecting MDBK cells.

To quantify the impact of sphingomyelinase and methyl-β-cyclodextrin treatments on BVDV infectivity, MDBK cells (10000 cells per well of 96-well plate) were infected for 1 h at 37°C with 100 μl of treated or control virus. The virus was removed and the cells were overlaid with 100 μl of fresh culture medium. Cells were fixed 15 h later with 3% paraformaldhehyde in PBS and processed for immunofluorescent detection of NS3. Nuclei were stained with DAPI. Samples were observed with a Zeiss Axiophot microscope equipped with a 10 X magnification objective. Fluorescent signals were collected with a Coolsnap ES camera (Photometrix). For quantification, images of randomly picked areas from each well were recorded and processed using ImageJ software. The total number of cells was obtained from DAPI-labeled nuclei. NS3-positive cells were counted as infected cells. The infection was scored as ratio of infected cells to total cells.

To assess the impact of sphingomyelinase and methyl-β-cyclodextrin treatments on BVDV structure, purified BVDV eluted from the cellulose-sulfate column was incubated at 37°C with sphingomyelinase (1 U/ml) or methyl-β-cyclodextrin (2.5 or 5 mg/ml) or the same volume of PBS (sphingomyelinase and methyl-β-cyclodextrin solvent). Treated virus was then transferred on ice and kept at 4°C until cryo-electron microscopy analysis. A small volume was removed for verifying the impact of treatments on infectivity.

### Analysis of detergent-resistant membranes of infected cells and viral particles

Lipid rafts were isolated by standard procedures. Infected MDBK cells were grown 150-mm dish to reach confluence. Cells were rinsed with cold PBS and lysed in 1 ml of TNE containing 0.8% Triton X-100, and supplemented with protease inhibitors (Roche). Lysates were scraped off the dish with a cell lifter, the dish was rinsed with 1 ml of the same buffer at 4°C, and the lysate was homogenized in a dounce homogenizer. The extract was finally brought up to 40% sucrose in a final volume of 4 ml and sequentially overlaid with 5 ml of 30% sucrose and 3 ml of 5% sucrose. Gradients were centrifuged for 18 h at 40,000 rpm at 4°C in a Beckman SW41 rotor. Fractions (1 ml) were collected from the bottom of the gradient and immediately supplemented with 20μl of a fresh 50X solution of protease inhibitors. Aliquots of each fraction were analyzed by immunoblotting.

## Supporting Information

S1 Text Supplementary methods and table A. (DOCX)

S1 Table Results of lipid quantification. (XLSX)

S1 Fig Gallery of cryo-electron microscopy images of purified BVDV fractions. (PDF)

S2 Fig Comparative analysis of purified BVDV and control fractions. Cryo-electron microscopy analysis of purified BVDV (top panels) and a corresponding control fraction purified from non-infected MDBK cells (bottom panels). Bars, 1 μm (left panels) or 100 nm (right panels).
(PDF)

S3 Fig Immunogold electron microscopy of purified BVDV particles. Purified BVDV particles were labeled with (top panels) or without (bottom panels) anti-E2 mAbs, before negative staining. Arrowheads indicate colloidal gold particles bound on viral particles. Bars, 100 nm.
(PDF)

S4 Fig Neutralization of purified and unpurified BVDV with anti-E2 mAbs. Error bars correspond to SDs (n = 4).
(PDF)

S5 Fig Cryo-electron microscopy analysis of flow-through and elution fractions of cellulose-sulfate chromatography. (A-D) Flow-through fraction. (E, F) 0.4 M NaCl elution fraction. Bars, 500 nm (A) or 100 nm (B,D). Bars, 500 nm (A) or 100 nm (B).
(PDF)

S6 Fig Cryo-electron microscopy analysis of purified BVDV particles of cytopathic strain NADL. Bars, 500 nm (top panels) or 100 nm (bottom panels).
(PDF)

S7 Fig Cryo-electron microscopy images of purified NADL particles incubated at pH 5.1 in the presence of 10 mM DTT. (PDF)

S8 Fig Immunoblot analysis of E2 under non-reduced condition in NADL particles purification samples. (PDF)

S9 Fig Identification of E2 by mass spectrometry. (A) Annotated mass spectrum of trypsin digest of ~55 kDa fraction. Peptides assigned to E2 are indicated. (B) Protein view, peptides matching the sequence are represented as gray bars,
(PDF)

S10 Fig Lipid composition of purified BVDV and control fractions. The content of individual lipid classes of purified BVDV (uncorrected for background) and control fractions similarly purified from non-infected cells are indicated (A) in absolute amounts within the samples and (B) in relative amounts. Glycerophospholipids: phosphatidic acid (PA), phosphatidylcholine/-ethanolamine/-glycerol/-inositol/-serine (PC/PE/PG/PI/PS), and ether linked PC/PE (ePC/ePE). Sphingolipids: ceramide (Cer), sphingomyelin (SM) and hexosylceramide (HexCer). Sterols: cholesterol (Chol). Storage neutral lipids: cholesteryl esters (CE), diacylglycerol (DAG) and triacylglycerol (TAG). Error bars correspond to SDs (n = 2).
(PDF)

S11 Fig Species of main phospholipids and sphingolipids of BVDV and MDBK cells. Saturation as a number of double bonds in both fatty acyl chains in (A) phosphatidylethanolamine (PE), (B) ether linked PE (ePE), (C) ethanolamine plasmalogen (pl-PE), (D) ether linked phosphatidylcholine (ePC), (E) phosphatidylserine (PS) (F) phosphatidylinositol (PI), (G) sphingomyelin (SM) and (H) hexosylceramide (HexCer) are indicated. Error bars correspond to SDs (n = 3).
(PDF)

S12 Fig Control experiments of sphingomyelinase and methyl-β-cyclodextrin treatments. (A) MDBK cells were infected with BVDV pre-treated with sphingomyelinase as explained in the legend of Fig 9 (blue bars), incubated with SMase for 2 h and infected with untreated BVDV (red bars), incubated with untreated BVDV and then incubated with SMase for 2 h, or infected with BVDV in the presence of SMase without preincubation of BVDV and SMase. (B) Partially purified BVDV was incubated or not at 37°C for 1 h with 5mg/ml methyl-β-cyclodextrin (MBCD), diluted 10,000 times and used to infect MDBK cells. The number of infected cells was measured using an immunofluorescence assay at 15 hpi and standardized to the number of cells infected with untreated virus..
(PDF)

S13 Fig Cryo-electron microscopy analysis purified BVDV treated with sphingomyelinase or methyl-β-cyclodextrin. Purified BVDV was incubated for 1 h at 37°C with (A) no treatment, (B) 1U/ml SMase, (C) 2.5 mg/ml MBCD, or (D) 5 mg/ml MBCD, and analyzed by cryo-electron microscopy. Insets, low magnification image. Bars, 500 nm (insets) or 100 nm (main image).
(PDF)

S14 Fig Cryo-electron microscopy image of BVDV-derived aggregates induced by 5mg/ml methyl-β-cyclodextrin. Purified BVDV was incubated for 1 h at 37°C with 5 mg/ml MBCD, and analyzed by cryo-electron microscopy. Inset, high magnification image. Bars, 500 nm (main image) or 100 nm (inset).
(PDF)
