# Supplementary material for: Morphology and Molecular Composition of Purified Bovine Viral Diarrhea Virus Envelope
Source: PLoS Pathog. 2016 Mar 3;12(3):e1005476. doi: 10.1371/journal.ppat.1005476 (PMC4777508; doi:10.1371/journal.ppat.1005476)
Supplement: S1 Text — (DOCX) [file ppat.1005476.s001.docx]

**SUPPORTING MATERIALS AND METHODS**

***Immunogold labeling of BVDV particles***

Immunoglod labeling was performed on virus sample (5 µl) deposited on carbon coated nickel grids submitted to a glow discharge (Elmo, Cordouan). Briefly, samples were labelled with anti-E2 antibodies (1:50) followed by the protein-A coupled to 10 nm colloidal gold particles (CMC Utrecht). After a fixation step with glutaraldehyde , sample were finally negatively stained using 2% uranyl acetate. Grids were observed on a FEI CM120 electron microscope operating at 120 kV. Images were recorded with a 2k x 2k USC 1000 slow-scan CCD camera (Gatan).

***Neutralization with anti-E2 antibodies***

Unpurified and purified BVDV were incubated at room temperature for 20 minutes in 100µl of MDBK culture medium with 10-fold dilution series of a mixture of anti-E2 mAbs WB166 and WB214, and then incubated for 1 h at 37°C with MDBK cells in P96 plate. Inoculate were removed and new culture medium was added. Cells were fixed at 16 hpi and processed for immunofluorescence detection of NS3. Nuclei were labeled with DAPI. The infection was scored as percentage of infected cells.

***Acidic pH treatment of BVDV particles***

Purified BVDV particles of NADL strain were diluted in a buffer at pH 5.1 containing 135 mM NaCl, 15 mM sodium citrate, 10 mM MES, 5 mM HEPES, 1 mM EDTA and 10 mM DTT, and then observed by cryo-electron microscopy.

**S2 TABLE. Primers used for RT-PCR**

reverse transcription:

5’-ATCGACATCACAGTAAGGGGAAGC-3’

5’-TGGGTCACGCCTAGCTATGATTAAACC-3’

PCR:

5’-ATTGCTACTAAAACTCTC-3’

5’-GGTATGATGGACGCAAGT-3’

5’-AAGCCACCAGAATCACGC-3’

5’-TGGTTTGGAGCATATGCG-3’

5’-GAAAGTGCCAGACAAGGG-3’

5’-GGGGACACAGTCGAAATG-3’

5’-ACTTTGCATTCGAGGGCC-3’

5’-GACCAAGGGTACTGGGGG-3’

**.**
