# Supplementary figures and images for: Morphology and Molecular Composition of Purified Bovine Viral Diarrhea Virus Envelope
Source: PLoS Pathog. 2016 Mar 3;12(3):e1005476. doi: 10.1371/journal.ppat.1005476 (PMC4777508; doi:10.1371/journal.ppat.1005476)

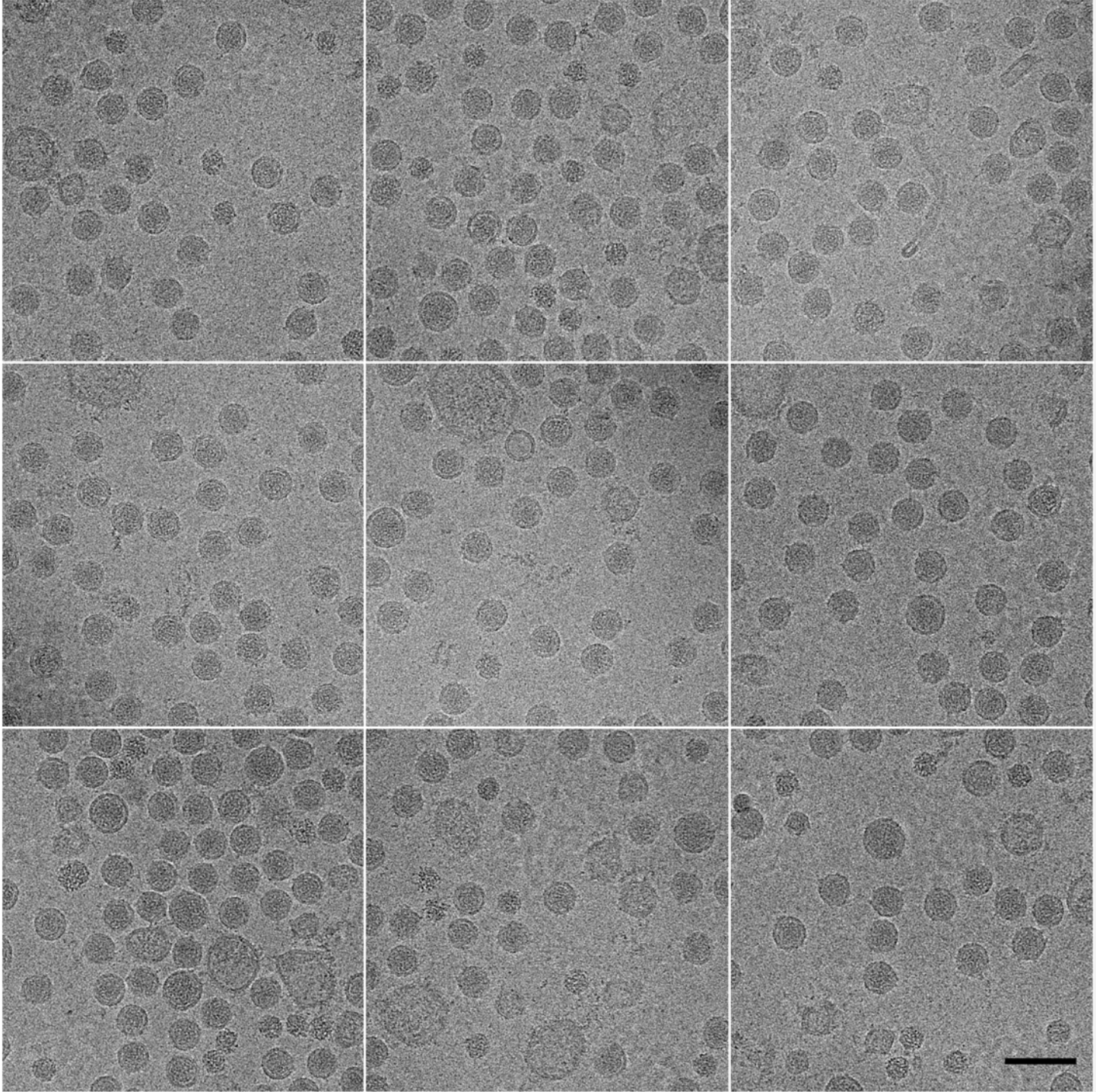

Supplement: S1 Fig — (PDF) [file ppat.1005476.s003.pdf]

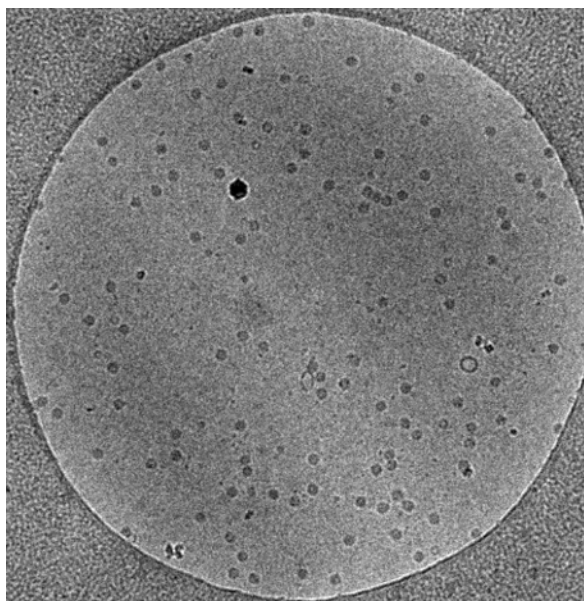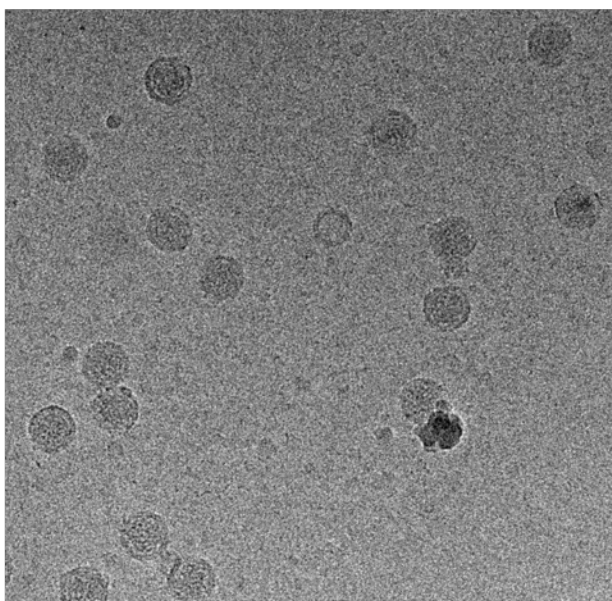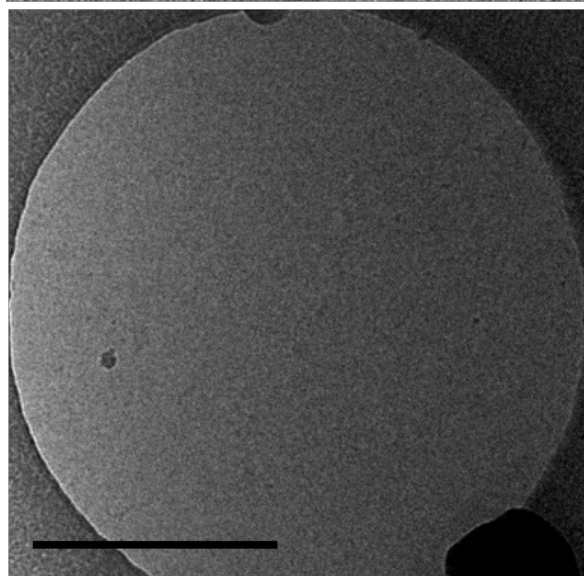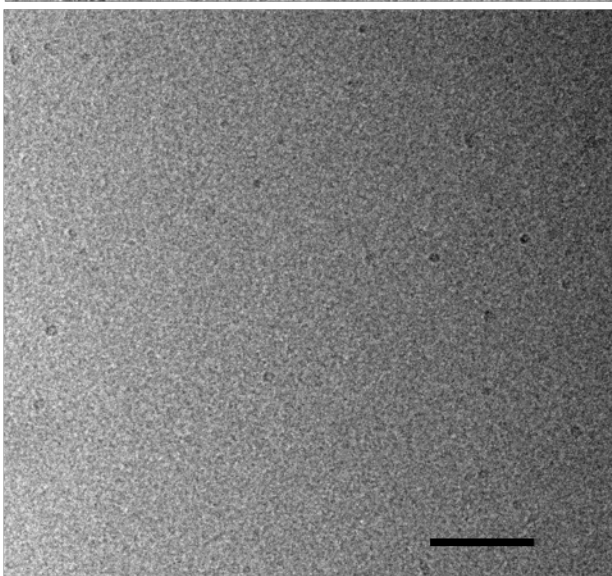

Supplement: S2 Fig — Cryo-electron microscopy analysis of purified BVDV (top panels) and a corresponding control fraction purified from non-infected MDBK cells (bottom panels). Bars, 1 μm (left panels) or 100 nm (right panels). (PDF) [file ppat.1005476.s004.pdf]

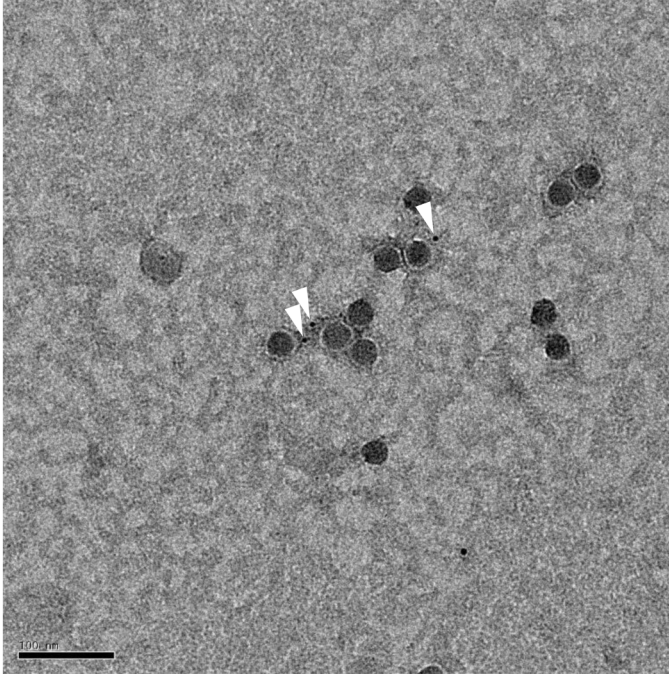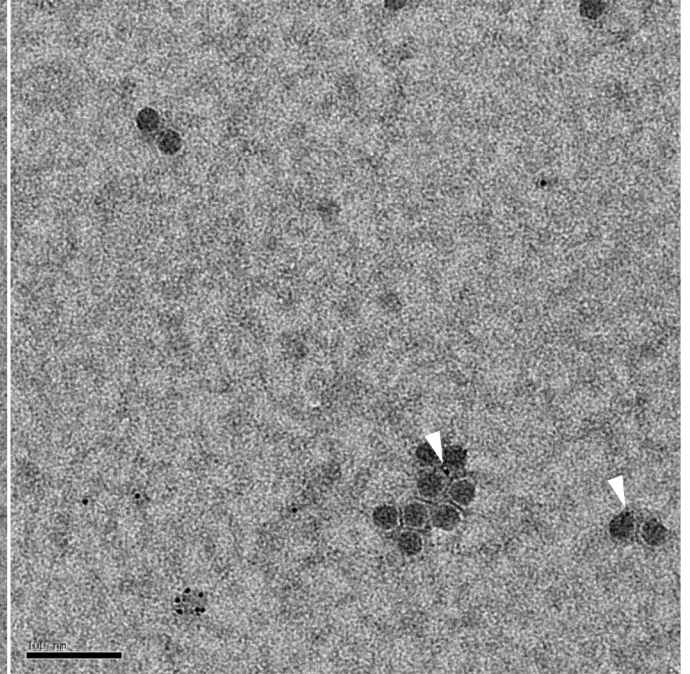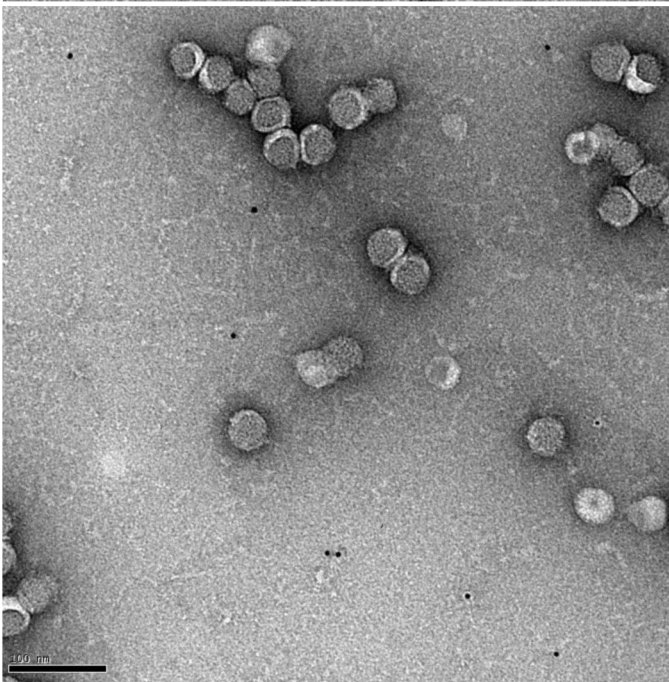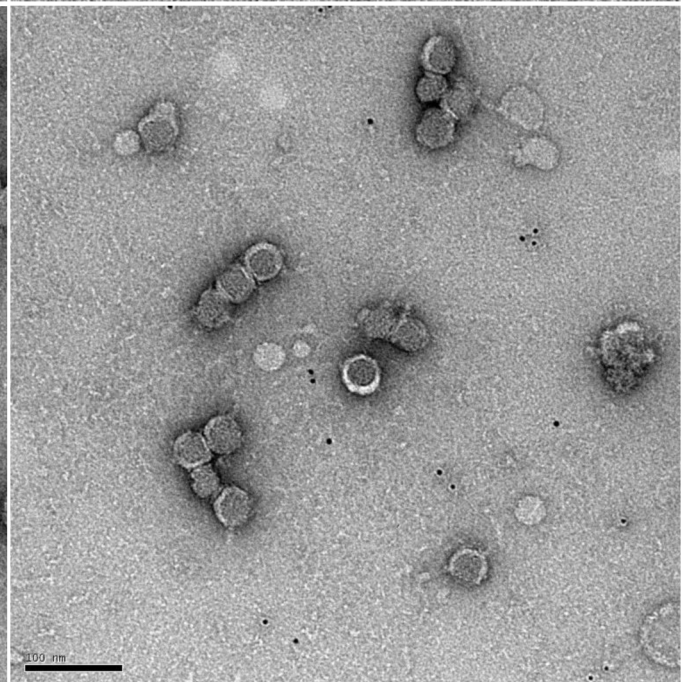

Supplement: S3 Fig — Purified BVDV particles were labeled with (top panels) or without (bottom panels) anti-E2 mAbs, before negative staining. Arrowheads indicate colloidal gold particles bound on viral particles. Bars, 100 nm. (PDF) [file ppat.1005476.s005.pdf]

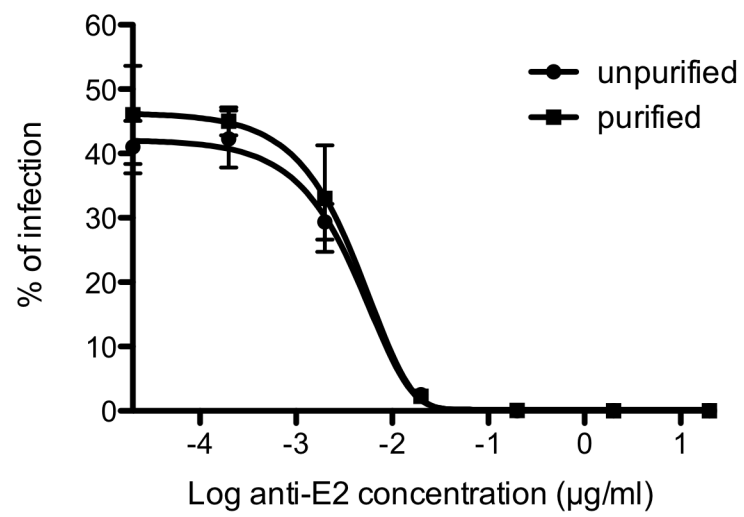

Supplement: S4 Fig — Error bars correspond to SDs (n = 4). (PDF) [file ppat.1005476.s006.pdf]

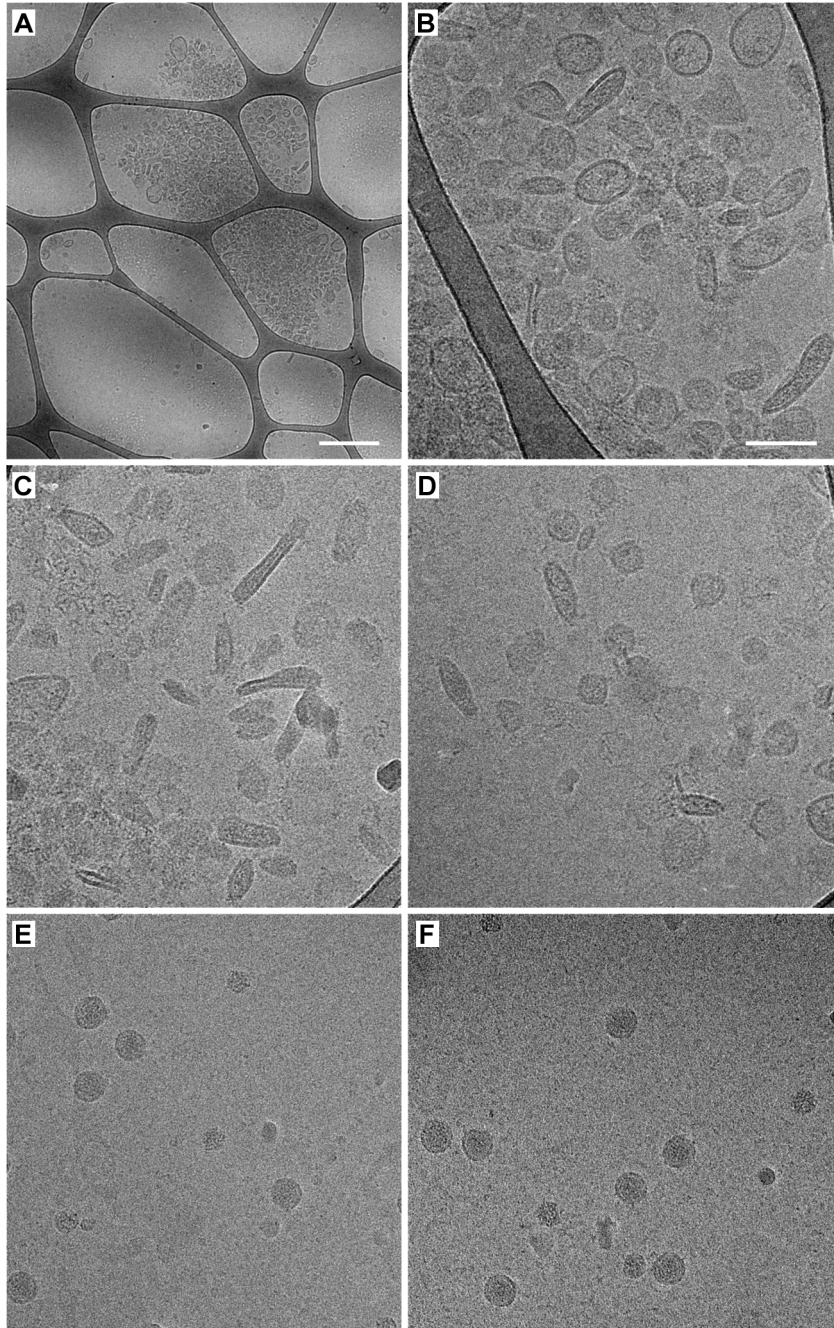

Supplement: S5 Fig — (A-D) Flow-through fraction. (E, F) 0.4 M NaCl elution fraction. Bars, 500 nm (A) or 100 nm (B,D). Bars, 500 nm (A) or 100 nm (B). (PDF) [file ppat.1005476.s007.pdf]

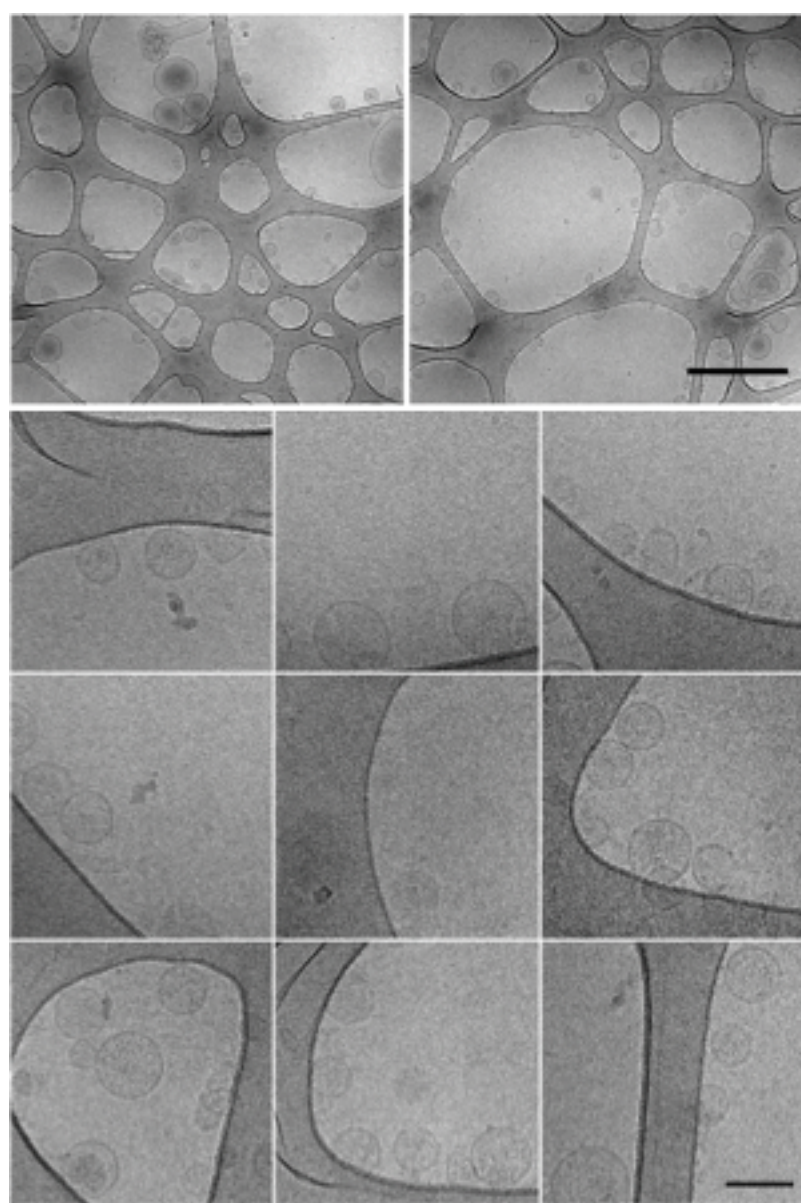

Supplement: S6 Fig — Bars, 500 nm (top panels) or 100 nm (bottom panels). (PDF) [file ppat.1005476.s008.pdf]

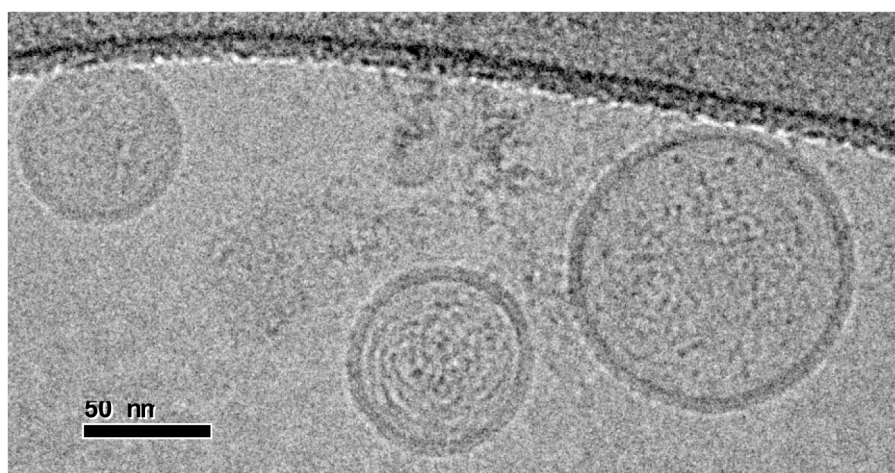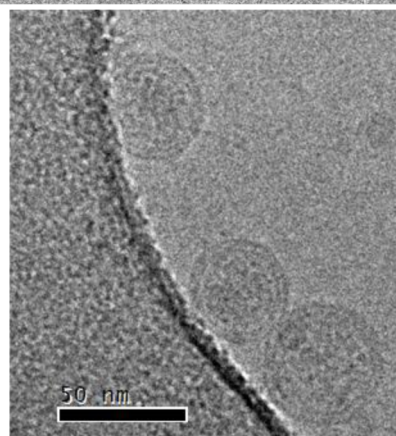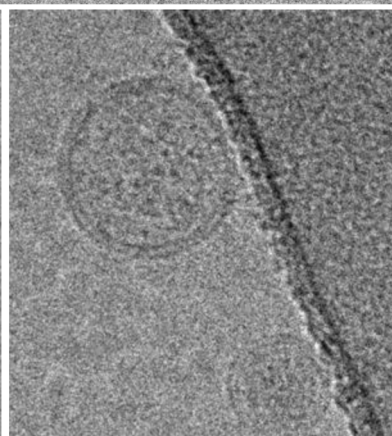

Supplement: S7 Fig — (PDF) [file ppat.1005476.s009.pdf]

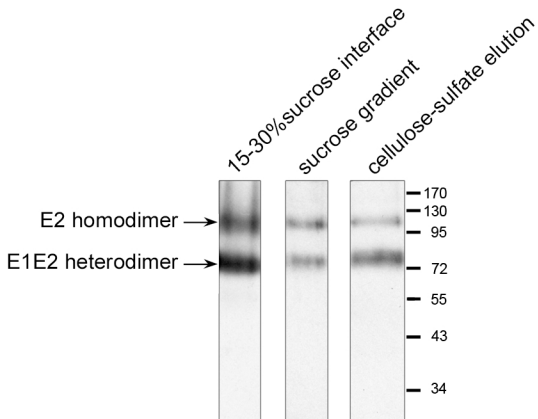

Supplement: S8 Fig — (PDF) [file ppat.1005476.s010.pdf]

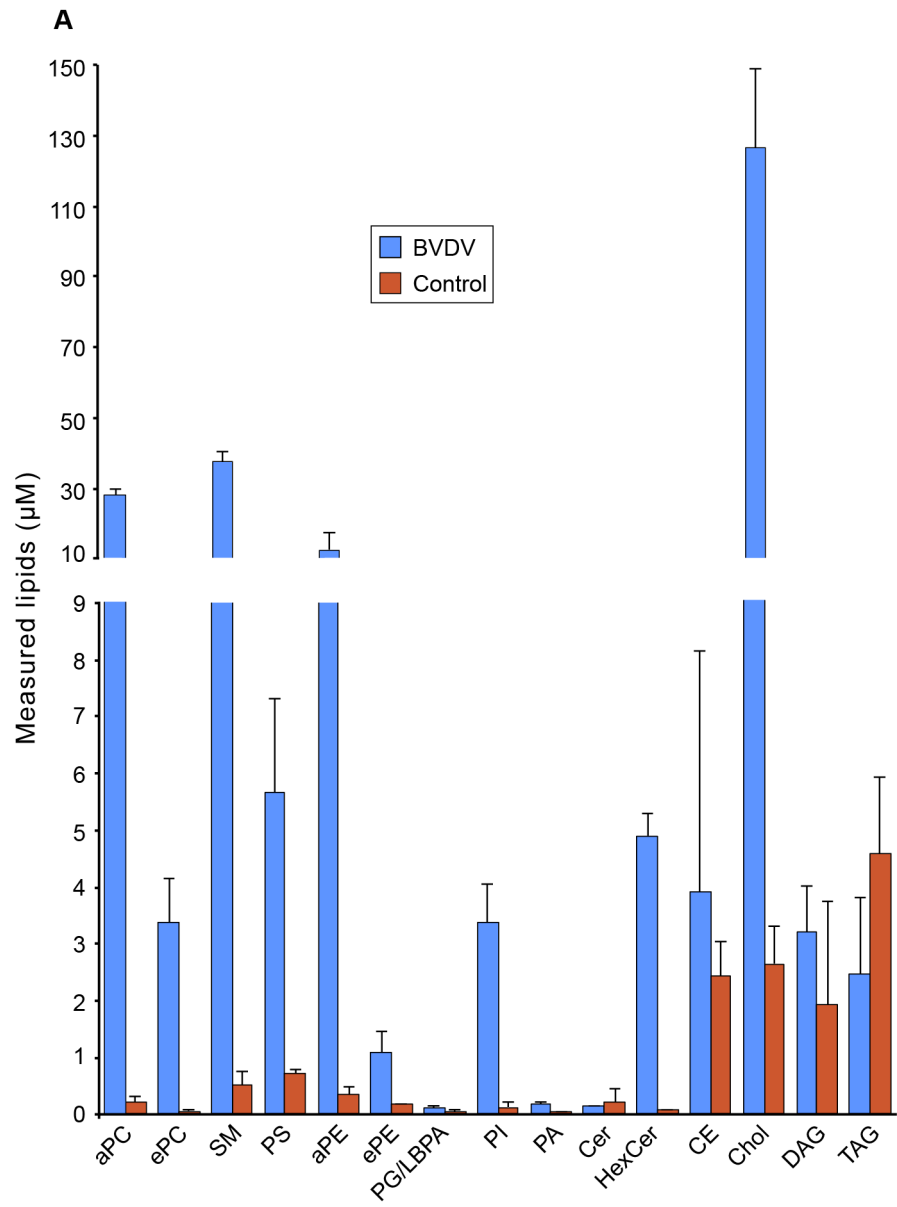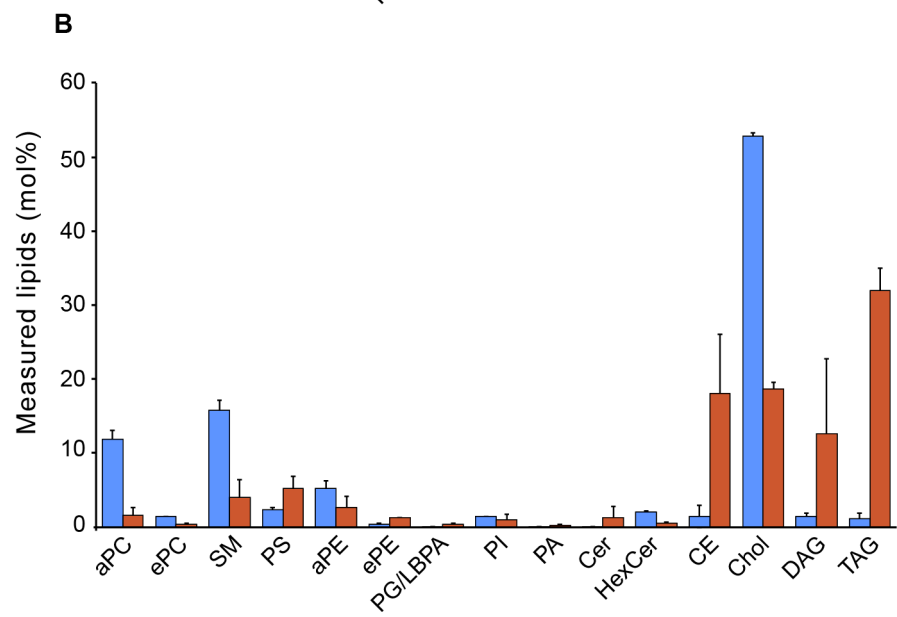

Supplement: S10 Fig — The content of individual lipid classes of purified BVDV (uncorrected for background) and control fractions similarly purified from non-infected cells are indicated (A) in absolute amounts within the samples and (B) in relative amounts. Glycerophospholipids: phosphatidic acid (PA), phosphatidylcholine/-ethanolamine/-glycerol/-inositol/-serine (PC/PE/PG/PI/PS), and ether linked PC/PE (ePC/ePE). Sphingolipids: ceramide (Cer), sphingomyelin (SM) and hexosylceramide (HexCer). Sterols: cholesterol (Chol). Storage neutral lipids: cholesteryl esters (CE), diacylglycerol (DAG) and triacylglycerol (TAG). Error bars correspond to SDs (n = 2). (PDF) [file ppat.1005476.s012.pdf]

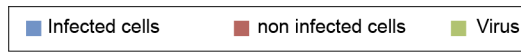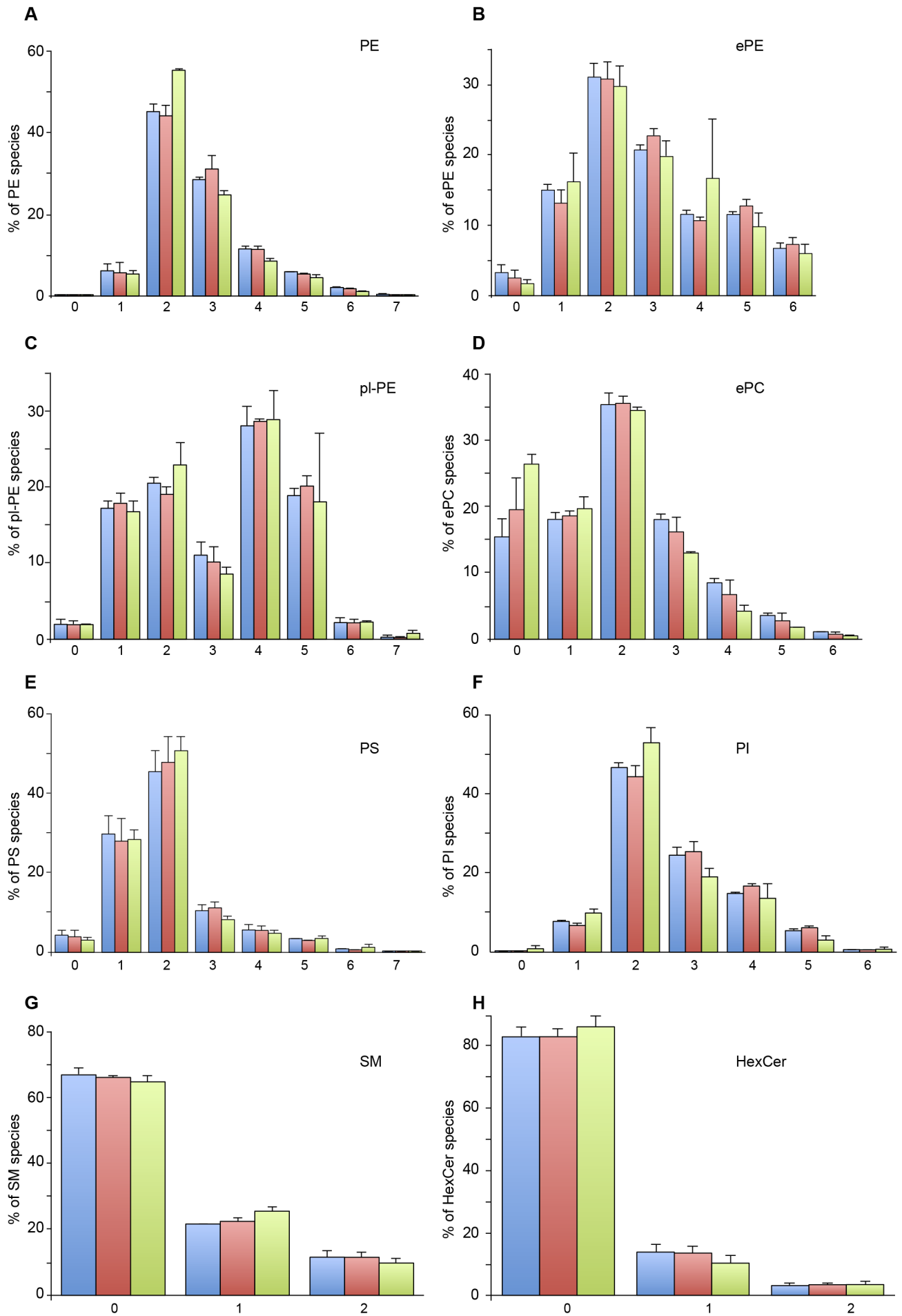

Supplement: S11 Fig — Saturation as a number of double bonds in both fatty acyl chains in (A) phosphatidylethanolamine (PE), (B) ether linked PE (ePE), (C) ethanolamine plasmalogen (pl-PE), (D) ether linked phosphatidylcholine (ePC), (E) phosphatidylserine (PS) (F) phosphatidylinositol (PI), (G) sphingomyelin (SM) and (H) hexosylceramide (HexCer) are indicated. Error bars correspond to SDs (n = 3). (PDF) [file ppat.1005476.s013.pdf]

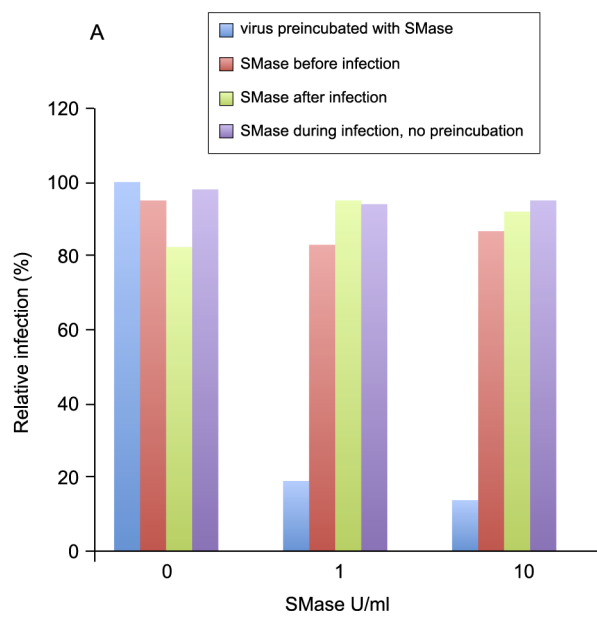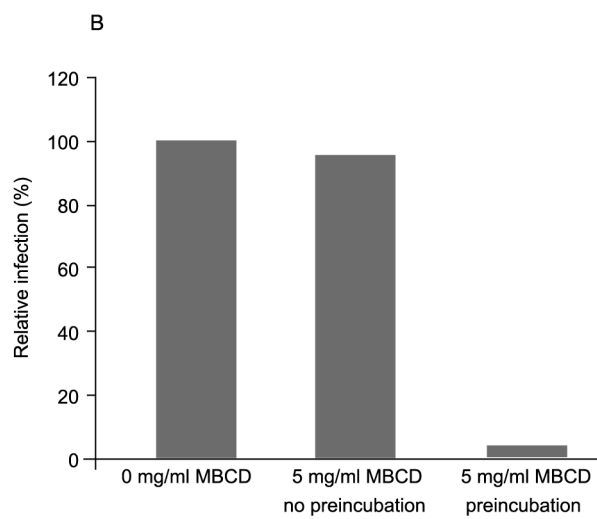

Supplement: S12 Fig — (A) MDBK cells were infected with BVDV pre-treated with sphingomyelinase as explained in the legend of Fig 9 (blue bars), incubated with SMase for 2 h and infected with untreated BVDV (red bars), incubated with untreated BVDV and then incubated with SMase for 2 h, or infected with BVDV in the presence of SMase without preincubation of BVDV and SMase. (B) Partially purified BVDV was incubated or not at 37°C for 1 h with 5mg/ml methyl-β-cyclodextrin (MBCD), diluted 10,000 times and used to infect MDBK cells. The number of infected cells was measured using an immunofluorescence assay at 15 hpi and standardized to the number of cells infected with untreated virus.. (PDF) [file ppat.1005476.s014.pdf]

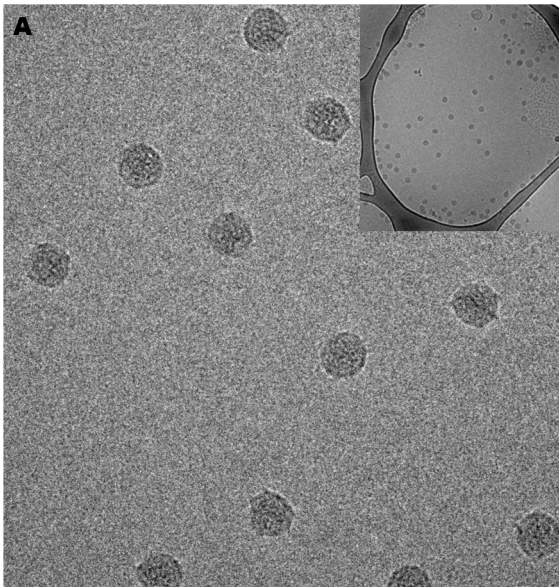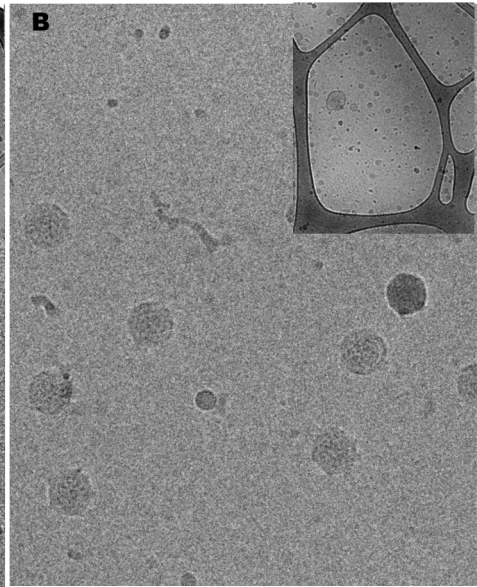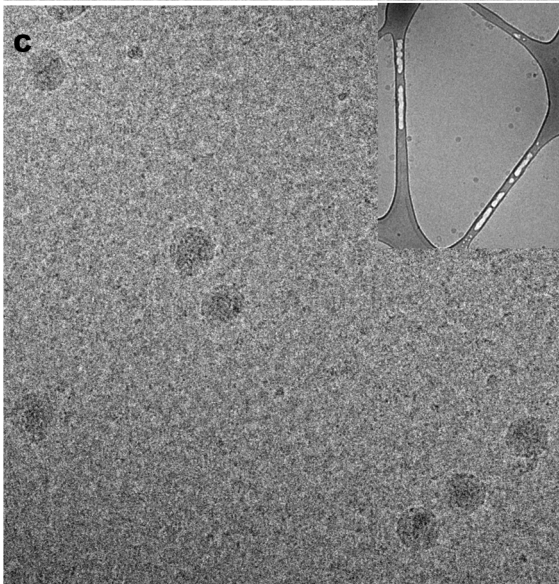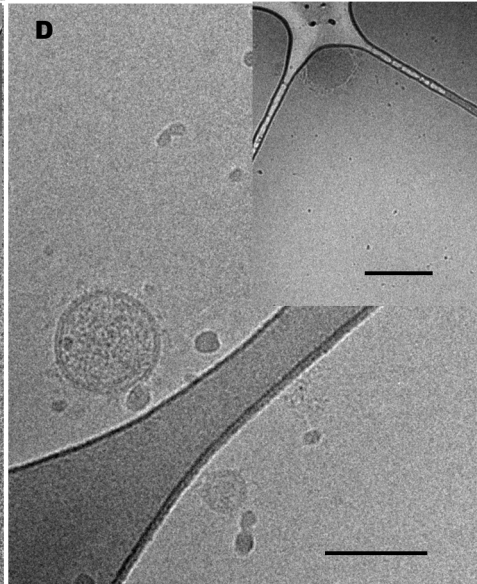

Supplement: S13 Fig — Purified BVDV was incubated for 1 h at 37°C with (A) no treatment, (B) 1U/ml SMase, (C) 2.5 mg/ml MBCD, or (D) 5 mg/ml MBCD, and analyzed by cryo-electron microscopy. Insets, low magnification image. Bars, 500 nm (insets) or 100 nm (main image). (PDF) [file ppat.1005476.s015.pdf]

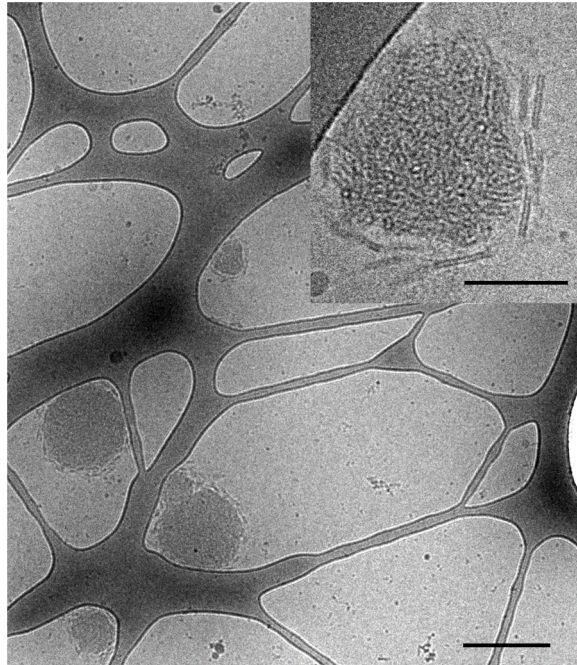

Supplement: S14 Fig — Purified BVDV was incubated for 1 h at 37°C with 5 mg/ml MBCD, and analyzed by cryo-electron microscopy. Inset, high magnification image. Bars, 500 nm (main image) or 100 nm (inset). (PDF) [file ppat.1005476.s016.pdf]
