# Supplementary material for: Morphology and Molecular Composition of Purified Bovine Viral Diarrhea Virus Envelope
Source: PLoS Pathog. 2016 Mar 3;12(3):e1005476. doi: 10.1371/journal.ppat.1005476 (PMC4777508; doi:10.1371/journal.ppat.1005476)
Supplement: S9 Fig — (A) Annotated mass spectrum of trypsin digest of ~55 kDa fraction. Peptides assigned to E2 are indicated. (B) Protein view, peptides matching the sequence are represented as gray bars, (PDF) [file ppat.1005476.s011.pdf]

A

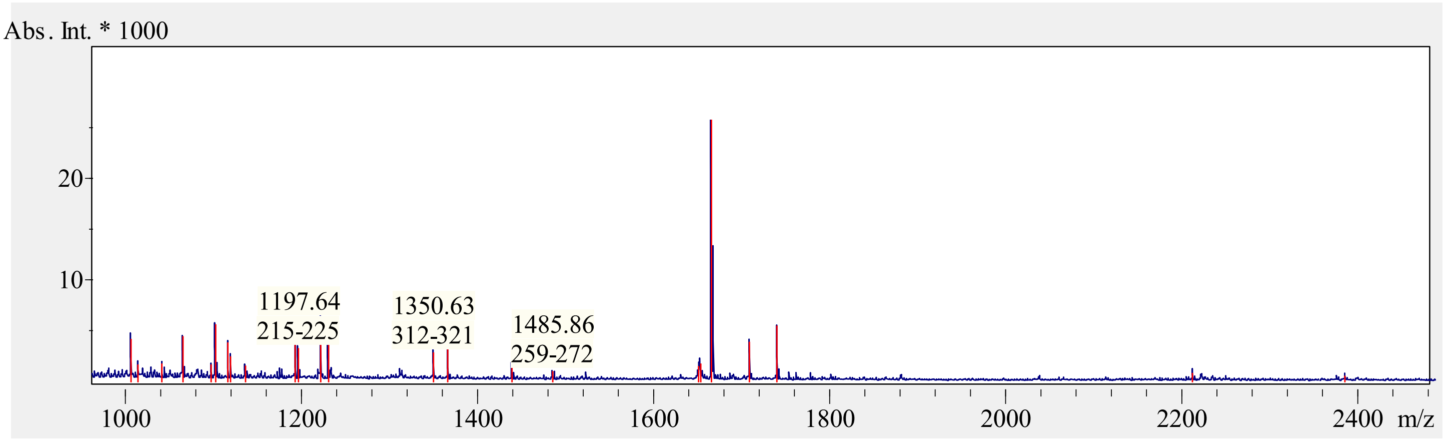
Abs. Int. * 1000
20
10
1197.64
215-225
1350.63
312-321
1485.86
259-272
1000
1200
1400
1600
1800
2000
2200
2400
m/z

B

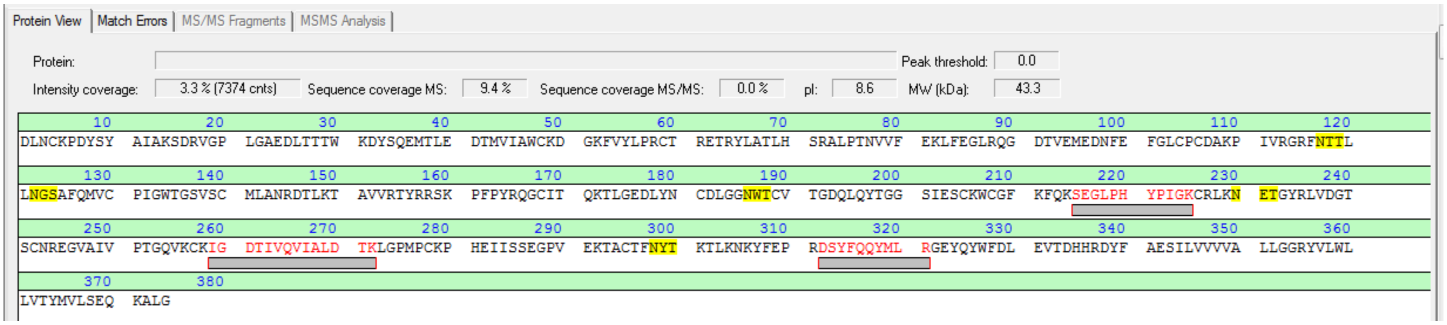
Protein View
Match Errors
MS/MS Fragments
MSMS Analysis
Protein:
Peak threshold: 0.0
Intensity coverage: 3.3 % (7374 cnts)
Sequence coverage MS: 9.4 %
Sequence coverage MS/MS: 0.0 %
pI: 8.6
MW (kDa): 43.3
10 20 30 40 50 60 70 80 90 100 110 120
DLNCKPDYSY AIAKSDRVGP LGAEDLTTTW KDYSQEMTLE DTMVIAWCKD GKFVYLPRCT RETRYLATLH SRALPTNVVF EKLFEGLRQG DTVEMEDNFE FGLCPCDAKP IVRGRFNTTL
130 140 150 160 170 180 190 200 210 220 230 240
LNGSAFQMVC PIGWTGSVSC MLANRDTLKT AVVRTYRRSK PFPYRQGCIT QKTLGEDLYN CDLGGNWTCV TGDQLQYTGG SIESCKWCGF KFQKSEGLPH YPIGKCRLKN ETGYRLVDGT
250 260 270 280 290 300 310 320 330 340 350 360
SCNREGVAIV PTGQVKCKIG DTIVQVIALD TKLGPMPCKP HEIISSEGPV EKTACTFNYT KTLKNKYFEP RDSYFQQYML RGEYQYWFDL EVTDHHRDYF AESILVVVVA LLGGRYVLWL
370 380
LVTYMVLSEQ KALG
